# Novel Tetraphenolic Porphyrazine Capable of MRSA Photoeradication

**DOI:** 10.3390/molecules30153069

**Published:** 2025-07-22

**Authors:** Wojciech Szczolko, Eunice Zuchowska, Tomasz Koczorowski, Michal Kryjewski, Jolanta Dlugaszewska, Dariusz T. Mlynarczyk

**Affiliations:** 1 Chair and Department of Chemical Technology of Drugs, Poznan University of Medical Sciences, Rokietnicka 3, 60-806 Poznan, Poland; eunicezuchowska@gmail.com (E.Z.); tkoczorowski@ump.edu.pl (T.K.); mlynarczykd@ump.edu.pl (D.T.M.); 2 Chair and Department of Inorganic and Analytical Chemistry, Poznan University of Medical Sciences, Rokietnicka 3, 60-806 Poznan, Poland; mkryjewski@ump.edu.pl; 3 Chair and Department of Genetics and Pharmaceutical Microbiology, Poznan University of Medical Sciences, Rokietnicka 3, 60-806 Poznan, Poland; jdlugasz@ump.edu.pl

**Keywords:** photodynamic antimicrobial chemotherapy, photodynamic inactivation, porphyrazine, pyrrole, silane, singlet oxygen

## Abstract

This work presents the synthesis, characterization and evaluation of physicochemical and biological properties of two new aminoporphyrazine derivatives bearing magnesium(II) cations in their cores and peripheral pyrrolyl groups. The synthesis was carried out in several stages, using classical methods and the Microwave-Assisted Organic Synthesis (MAOS) approach. The obtained compounds were characterized using spectral techniques: UV-Vis spectrophotometry, mass spectrometry, ^1^H and ^13^C NMR spectroscopy. The porphyrazine derivatives were tested for their electrochemical properties (CV and DPV), which revealed four redox processes, of which in compound **7** positive shifts of oxidation potentials were observed, resulting from the presence of free phenolic hydroxyl groups. In spectroelectrochemical measurements, changes in UV-Vis spectra associated with the formation of positive-charged states were noted. Photophysical studies revealed the presence of characteristic absorption Q and Soret bands, low fluorescence quantum yields and small Stokes shifts. The efficiency of singlet oxygen generation (Φ_Δ_) was higher for compound **6** (up to 0.06), but compound **7**, despite its lower efficiency (0.02), was distinguished by a better biological activity profile. Toxicity tests using the *Aliivibrio fischeri* bacteria indicated the lower toxicity of **7** compared to **6**. The most promising result was the strong photodynamic activity of porphyrazine **7** against the Methicillin-resistant *Stapylococcus aureus* (MRSA) strain, leading to a more-than-5.6-log decrease in viable counts after the colony forming units (CFU) after light irradiation. Compound **6** did not show any significant antibacterial activity. The obtained data indicate that porphyrazine **7** is a promising candidate for applications in photodynamic therapy of bacterial infections.

## 1. Introduction

Over a century after the discovery of the first antibacterial drug—salvarsan, humanity is struggling with a growing number of bacteria that are drug-resistant [1,2]. This creates a huge problem for medicine, at the same time posing a significant threat to human life, as no appropriate therapy for such an eventuality exists. [3]. Therefore, alternative ways of fighting infectious diseases are sought for. In this context, reactive oxygen species (ROS) are of great interest for the discovery of new therapies that can be used in the treatment of, among others, infections with antibiotic-resistant bacteria [4]. A promising approach is photodynamic inactivation of microorganisms (PDI) [5]. This treatment method is based on the introduction of a non-toxic compound (a photosensitizer) into the pathogens—bacteria, fungi, viruses, or parasites [6]. Next, when the light of an appropriate wavelength reaches the photosensitizer, it is able to mediate the production of ROS [7]. This in turn causes oxidative stress, which cannot be balanced with antioxidant and repair mechanisms of the cell and thus leads to cell death. This in turn results in the inactivation of the microorganism, and consequently in the inhibition of the development of bacterial infection. Bacteria do not have an effective defense mechanism against damage caused by singlet oxygen. This results in high PDI efficiency when the compound generates a sufficient amount of ROS [8]. PDI is also not based on the mechanism of action of the drug with the receptor. The inactivating effect on the microorganism is the result of ROS action on subcellular organelles and macromolecules, such as proteins, lipids, and nucleic acids [9]. Such a mechanism of action minimizes the risk of resistance development, which is the biggest disadvantage of classical therapies [10].

An interesting aspect is that singlet oxygen-producing reactions are processes that are not dependent on diffusion, but rather on the rate constant of the bimolecular reaction and the local concentration of triplet oxygen [11]. It is not clear which type of ROS is more effective. The hydroxyl radical (HO^**•**^) damages the bacterial wall and singlet oxygen destroys structures within the cell. It is believed that the combination of these two types of ROS may be the most effective [12]. The compounds that are typically applied as photosensitizers for studying PDI are porphyrinoids (porphyrins, chlorins, porphyrazines, phthalocyanines), phenothiazinium salts (i.e., methylene blue), curcuminoids, or—more recently—nanomaterials [13,14,15,16,17,18,19].

Porphyrazines (Pzs) are synthetic analogs of naturally occurring porphyrins. They owe their characteristic features to the circular arrangement of four pyrrole rings bridged with aza-methine moieties, which form an aromatic macrocycle [20]. The physicochemical properties of these macrocycles may differ depending on which metal ion will be coordinated in the macrocyclic core [21]. The difference between porphyrazines and porphyrins, the azamethine bridges, causes a reduction in the size of the macrocyclic compound core [22,23]. The change of porphyrins to porphyrazines turns out to be extremely effective in various fields of chemistry or medicine, due to their unique physicochemical properties [24].

Porphyrazines have been of interest to researchers for years, and there is a constant effort to improve their physicochemical properties such as solubility or efficiency of generating ROS [25]. This would enable a more effective use of these compounds in various fields of medicine, e.g., in photodynamic therapy [26]. Porphyrazines could be used as photosensitizers [27] and as redox catalysts [28]. Potentially, photosensitizing Pzs can be used in cancer therapies [29] and with the treatment of antibiotic-resistant bacteria. However, to make this possible, their solubility must be improved and the level of singlet oxygen production must be maintained or even increased, as these are the most commonly reported obstacles preventing the full use of these molecules [30]. Properties of the porphyrazines may be altered by modifying their peripheral substituents to include appropriate functional groups or by introduction of various cations within the coordinating center [31]. Mentioned modifications cause an increase or decrease in the desired features. Alternatively, the drawbacks of intrinsic hydrophobicity may be countermeasured by embedding in liposomal carriers. This increases the effectiveness of the therapy because the compound penetrates biological membranes to a greater extent [22].

The aim of this work was to synthesize a pyrrolyl-substituted porphyrazine derivative with an increased hydrophilic character and to evaluate its properties. This was achieved by protecting the phenolic hydroxyl groups with a silan-based group that could be cleaved post-macrocyclization. For the obtained derivative, aggregation studies were performed, as well as singlet oxygen generation measurements, both crucial for the assessment of the potential use of the Pz in PDI. Next, microbiological evaluation was carried out to confirm the photoinactivation effect on methicillin-resistant *Staphylococcus aureus*. To extend the characterization of the synthesized porphyrazine, the electrochemical and spectroelectrochemical experiments were conducted to determine the redox activity of macrocyclic ligands.

## 2. Results

### 2.1. Synthesis and Characterization

**Scheme 1:**
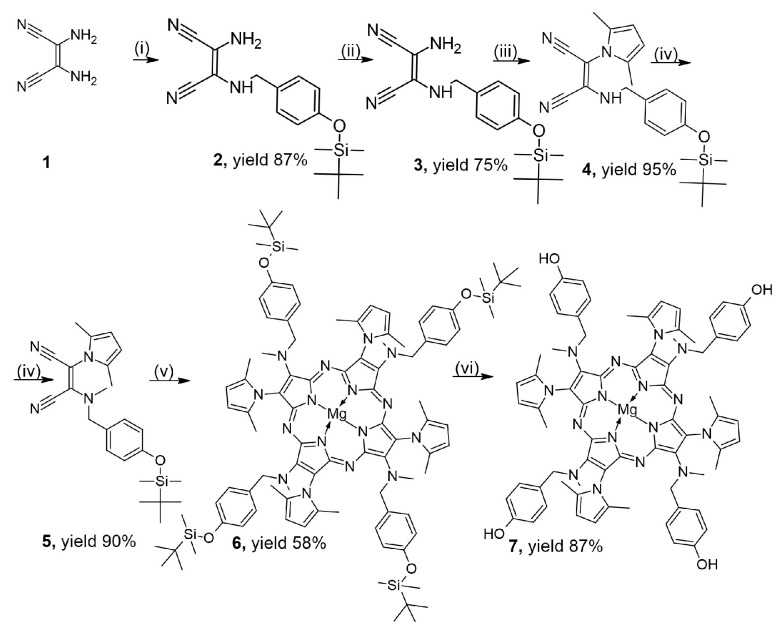
Synthetic pathway leading to porphyrazine **7**. Conditions and reagents: classical synthesis: (i) methanol, 4-(*tert*-butyldimethylsilyloxy)benzaldehyde, trifluoroacetic acid, rt 10 min; (ii) methanol, NaBH_4_, rt, 60 min; (iii) MAOS condition: methanol, hexan-2,5-dion, oxalic acid, 120 °C, 10 min (iv) MAOS condition: tetrahydrofuran, methyl iodide, Cs_2_CO_3_, 120 °C, 20 min; (v) MAOS condition: magnesium *tert*-butoxide, *n*-butanol, 180 °C, 10 min; (vi) tetrabutylammonium fluoride, tetrahydrofuran, 0 °C, 120 min.

Known 4-(*tert*-butyldimethylsilyloxy)benzaldehyde was synthetized according to a literature procedure [32]. Maleonitrile **2** was obtained in the condensation reaction between 4-(*tert*-butyldimethylsilyloxy)benzaldehyde with diaminomaleonitrile (DAMN) (**1**) in methanol with a catalytic amount of trifluoroacetic acid [30]. Next, maleonitrile **2** was subjected to reduction reaction with NaBH_4_ in methanol yielding derivative **3** [30]. Next, compound **3** was used in Paal-Knorr reaction using MAOS with hexano-2,5-dione in the presence of oxalic acid to give maleonitrile derivative **4** with 2,5-dimethylpyrrolyl group [33,34]. Maleonitrile **5**, as a mixture of *cis* and *trans* isomers, was obtained as the product of derivative **4** substituted with iodomethane in the presence of cesium carbonate in tetrahydrofuran (THF) using MAOS conditions [34]. Finally, maleonitrile derivative **5** was used in Linstead macrocyclization reaction, using MAOS conditions [35], with magnesium(II) *tert*-butoxide in *n*-butanol. The reaction mixture was heated at 180 °C for 10 min to obtain porphyrazine **6** in a 58% yield. The last synthetic step was the cleavage of the *tert*-butyldimethylsilane from **6** with tetrabutylammonium fluoride in THF for 1.5 h [36] to receive a dark green porphyrazine **7** in a 87% yield (Scheme 1).

The structure of all the new compounds was confirmed by mass spectrometry and NMR spectroscopy. Compounds **4** and **5** were obtained as a mixture of *cis* and *trans* isomers, which was observed as double signals in ^1^H and ^13^C NMR spectra (see Supporting Information). Based on the number of signals in both ^1^H and ^13^C NMR spectra of 6, it was found that only one regioisomer was formed, namely porphyrazine presenting *C_4h_* symmetry [37]. Such regioselective product formation was observed in our group before for pyrrolyl-substituted porphyrazines [27,38].

### 2.2. Electrochemical and Spectroelectrochemical Studies

**Figure 1:**
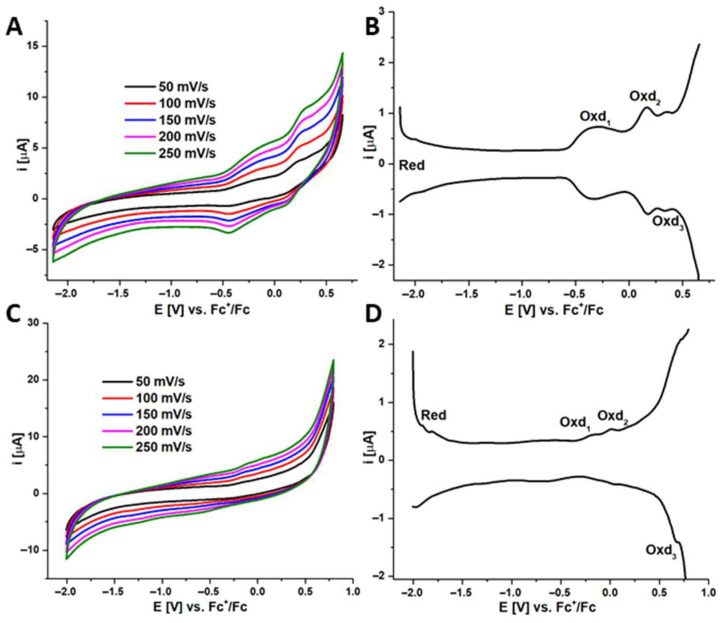
The CV voltammograms of **6** (**A**) and **7** (**C**) along with DPV voltammograms of **6** (**B**) and **7** (**D**). All measurements in DCM/0.1M TBAP. DPV parameters: modulation amplitude 20 mV, step rate 10 mV/s.

Given the chemical structure of macrocyclic ligands with the extended π-electron system, the electrochemical studies by means of cyclic and differential pulse voltammetries (CV and DPV, respectively) were conducted in deoxygenated DCM/0.1 M TBAP solution in a classic three-electrode system. The aim of these studies was to characterize synthesized macrocyclic ligands in terms of their potential electrochemical activity for future research, encompassing the sensing applicability and electrocatalytic tests. In the investigated electrochemical window (form −1.3 V to +1.2 V), in the cases of both porphyrazines **6** and **7**, four redox processes were observed—one reduction pair Pz^−1^/Pz and three oxidations—Pz/Pz^+1^; Pz^+1^/Pz^+2^, and Pz^+2^/Pz^+3^ (Figure 1). In the case of magnesium(II) complexes where the central metal cation in electrochemically inactive, the redox pairs can originate only from the macrocyclic ligand. However, due to the poor solubility of compound **7** in DCM, the voltammograms obtained did not reveal well-developed redox peaks (Figure 1C,D).

Based on DPV measurements, it was possible to record E_1/2_ values, showcased in Table 1. In both porphyrazines, the reduction processes were revealed at approx. −2.0 V. However, in terms of the oxidations, the first process (Oxd_1_) was shifted towards a more positive potential by approx. 0.13 V in **7**, compared to **6**. A more noticeable shift was observed in the case of the third oxidation process (Oxd_3_) where the potential was doubled in **7** compared to **6**. The presence of the third oxidation process in both compounds may stem from the electron removal from π-electrons of the benzyloxy substituents [34,39]. In addition, the shift of E_1/2_ of the Oxd_3_ process towards a more positive potential and its better development in **7** can be attributed to the susceptibility of the unprotected hydroxyl group to oxidation reaction [40]. When silylated in **6**, the process is hampered and only a tiny redox peak can be seen in voltammograms (Figure 1B). On the other hand, deprotected peripheral hydroxy groups in the case of **7** are prone to electron loss and, therefore, more pronounced signals can be observed (Figure 1D).

**Figure 2:**
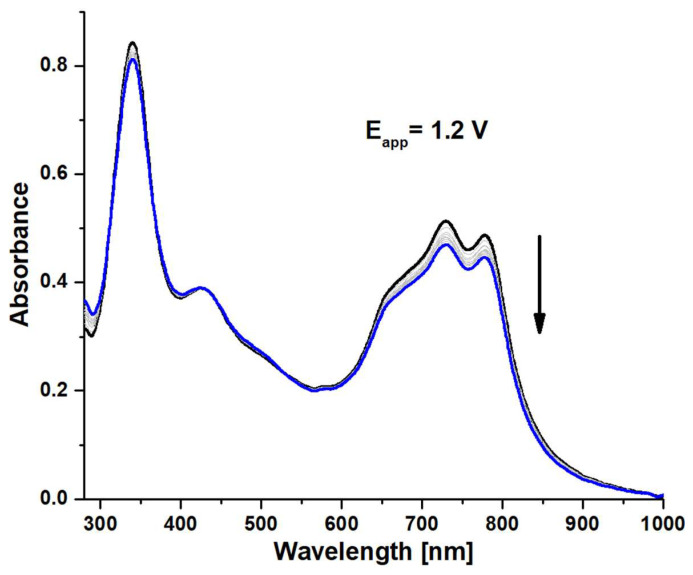
The UV-Vis spectra of **6** in DCM/0.1M TBAP at E_app_ = 1.2V.

In the spectroelectrochemical measurement, aimed to record the formation of anionic and cationic species of compounds **6** and **7**, noticeable changes in the UV-Vis spectra with application of a positive potential were observed. In the silylated porphyrazine **6**, these changes were observed at 1.2 V (Figure 2) with a decrease in Q band absorbance across time. This can be attributed to the aromaticity disruption of the macrocycle core and, therefore, to the formation of a cationic form of porphyrazine [Pz]^+^ [28].

**Figure 3:**
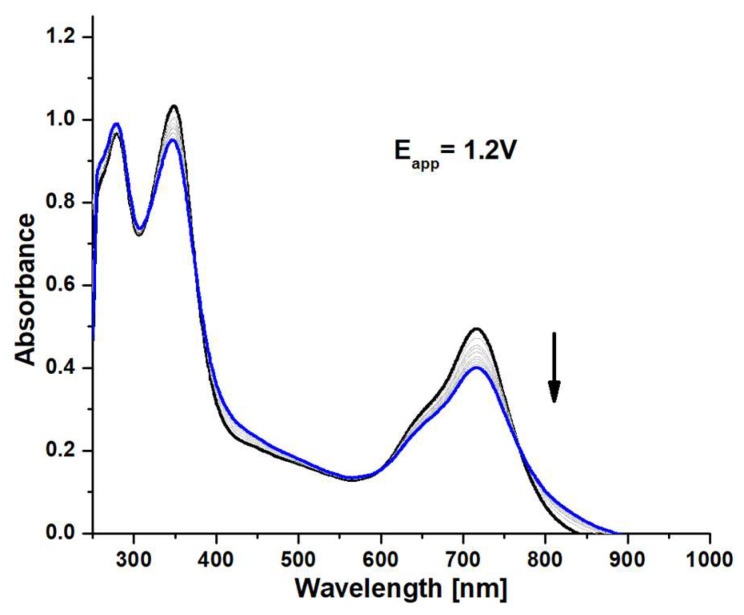
The UV–Vis spectra of **7** in DCM/0.1M TBAP at E_app_ = 1.2V.

A similar phenomenon was observed in **7,** where the Q band decline was also noticed at 1.2 V (Figure 3).

### 2.3. Absorption and Emission Properties

Photochemical properties of the Pzs were studied in two solvents, *N*,*N*-dimethylformamide (DMF) and dimethylsulfoxide (DMSO), whose main parameters are summarized in Table 2. Both Pzs show two main absorption bands—a Q band with the maxima in the range of 718–731 nm, and a Soret band (B band) with maxima around 350 nm. Absorbances of series of concentrations generally obey Beer–Lambert law (Figures S1–S4 Supporting Information), which suggest a lack of aggregation. The observed Q bands are results of the of π→π* transitions, namely S_0_→S_1_. As the molar absorption coefficients of Soret bands are higher than that of the Q band, we stipulate that this is a result of both S_0_→S_2_ transitions of the macrocycle core and the presence of the substituted phenyl groups.

Fluorescence quantum yields (Φ_F_) are very low for both macrocycles, though slightly higher for Pz **6**. Emission from the S_2_ state was not observed, unlike for some other pyrrole-bearing Pzs [39] (Figures S1–S8 Supporting Information).

### 2.4. Singlet Oxygen-Generation Measurements

**Figure 4:**
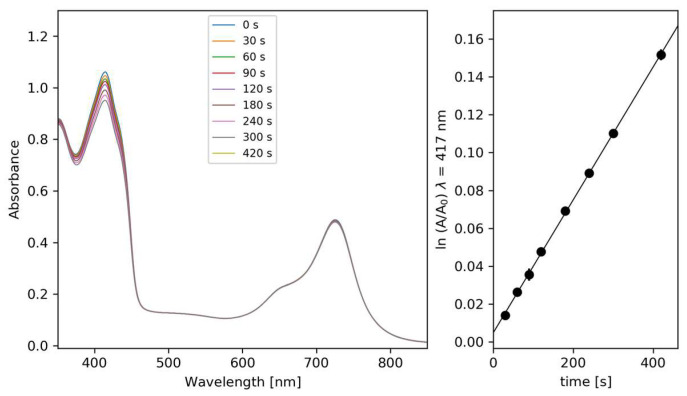
Changes in the UV-Vis spectrum, corresponding to different irradiation times for the mixture of Pz **6** and DPBF in DMF.

**Figure 5:**
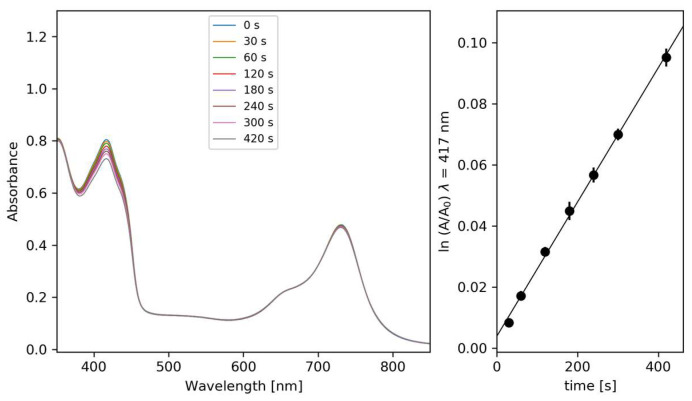
Changes in the UV-Vis spectrum, corresponding to different irradiation times for the mixture of Pz **6** and DPBF in DMSO.

**Figure 6:**
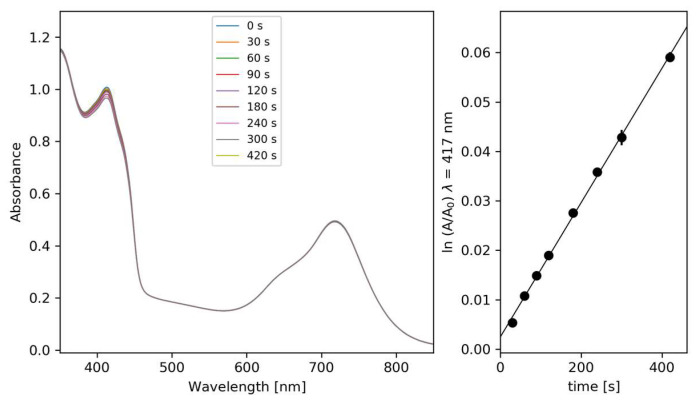
Changes in the UV-Vis spectrum, corresponding to different irradiation times for the mixture of Pz **7** and DPBF in DMF.

**Figure 7:**
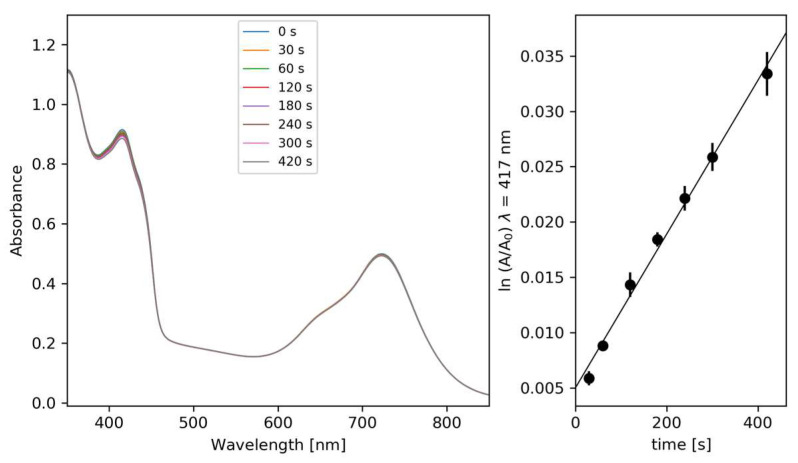
Changes in the UV-Vis spectrum, corresponding to different irradiation times for the mixture of Pz **7** and DPBF in DMSO.

Singlet oxygen-generation quantum yields (Φ_Δ_) of the Pzs were established using a comparative method, utilizing 1,3-diphenylizobenzofuran (DPBF) as a singlet oxygen quencher and zinc(II) phthalocyanine as a standard of the known Φ_Δ_ value (Table 2). Briefly, a solution of Pz and DPBF was irradiated with light of a wavelength corresponding to the Q band maximum, and DPBF decay was monitored by means of UV-Vis spectroscopy (Figure 4, Figure 5, Figure 6 and Figure 7). Measured values for Pz **7** are lower than that of Pz **6**. Previously reported Pzs bearing pyrrole-based substituents often generated singlet oxygen more efficiently, with the Φ_Δ_ parameter measuring up to 0.23 for the Pz bearing ester-pyrrole moieties [35] or 0.27 for the Pz decorated with dibromopyrrole [39]. However, Pzs **6** and **7** generate singlet oxygen more efficiently than analogs with three of four phenyl groups attached to the pyrrole ring.

### 2.5. Acute Toxicity Assessment Using Microtox Test

The newly synthesized porphyrazines, **6** and **7**, were subjected to the Microtox test. The basis of the test is the measurement of the bioluminescence of *Aliivibrio fischeri* bacteria before and after the addition of the tested sample [41]. When the bacteria come into contact with a toxic substance, the detected bioluminescence decreases in a linear manner.

**Figure 8:**
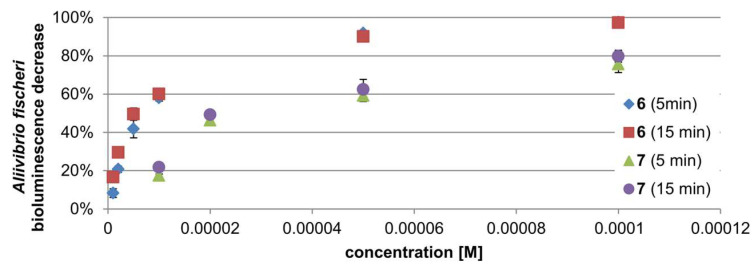
Changes in the *Aliivibrio fischeri* bioluminescence upon exposure to different concentrations of porphyrazines **6** and **7**.

The results of the performed experiments are summarized in Figure 8. As can be seen, the silane-protected macrocycle shows a higher toxic effect towards *A. fischeri* as compared to its free-hydroxyl counterpart. Interestingly, when considering the 20% decrease in bioluminescence values (a threshold used in Microtox analyses for the determination of toxicity [42]), a significant difference in concentration is needed to achieve such an effect—for **7** the toxicity was observed for concentrations exceeding 10^−5^ M, while for 6 at 2 × 10^−6^ M.

### 2.6. Photodynamic Inactivation Studies

The antibacterial activity of tested compounds was assessed against MRSA. This bacteria is among the most frequent causes of community- and hospital-acquired infections [43,44]. MRSA has developed resistance to most anti-staphylococcal antibiotics, which makes MRSA infections difficult to treat [45].

The effectiveness of tested compounds against MRSA was assessed using red light (730 nm) at an irradiance of 3 mW/cm^2^. The test was conducted over 60 min. and 90 min. The photodynamic activity of used Pzs was evaluated by comparing the number of bacteria in treated samples with that of untreated controls at each time point.

The results of our studies showed differences in the photodynamic activity of the compounds tested (Table 3). No antibacterial effect of **6** was observed on MRSA in either non-irradiated or irradiated samples, even when the time of irradiation was increased up to 90 min.

Conversely, porphyrazine **7** was able to photoinactivate MRSA to the detection limit of the method, and a reduction of >5.6 log_10_ in the bacterial viability was observed. In the case of non-irradiated samples, no effect was observed, which confirms the photodynamic mechanism of action. What is interesting is that the differences are not correlated to the singlet oxygen-generation quantum yields, which were almost the same for both derivatives. This suggests that the photodynamic effect has to be based either on a different ROS [46], or it proceeds via a different mechanism [47]. Alternatively, due to drastic differences in the polarity of the Pzs, the biological effect might be the result of different localizations of the Pzs within the bacterial cell [48].

No toxic effect on bacteria was demonstrated for methanol used to prepare the solutions of the tested porphyrazines (Table S1 in Supporting Information).

The above results explicitly demonstrated the excellent bactericidal photodynamic activity of porphyrazine **7**.

## 3. Materials and Methods

### 3.1. General Procedures

All reactions were conducted in oven-dried glassware under argon. All solvents were rotary evaporated at or below 50 °C. Microwave syntheses were performed on Anton-Paar Monowave 400 or Anton-Paar Monowave 400R. Solvents and all reagents were obtained from commercial suppliers and used without further purification. Melting points were obtained on a Stuart Bibby Scientific SMP10 apparatus and are uncorrected. Dry flash column chromatography was carried out on Merck silica gel 60, particle size 40–63 µm. Thin-layer chromatography (TLC) was performed on silica gel 60A F_254_ plates and visualized with UV (λ_max_ 254 or 365 nm). UV-Vis spectra were recorded on a JASCO V-770 spectrophotometer. HRMS spectra were recorded on Bruker Compact QTOF; monoisotopic mass values were calculated with https://www.chemcalc.org/ “URL acessed on 18 June 2025” [49]. ^1^H NMR, ^13^C NMR spectra were recorded using Bruker AvanceCore 400 at the Center of Innovative Pharmaceutical Technology at the Poznan University of Medical Sciences. Chemical shifts (δ) are quoted in parts per million (ppm) and are referred to as a residual solvent peak [50]. Coupling constants (*J*) are quoted in Hertz (Hz). The abbreviations s, bs, d, t, and m refer to singlet, broad singlet, doublet, triplet, and multiplet, respectively.

### 3.2. Synthesis of Derivatives **2**–**7**

2-Amino-3-[4-(*tert*-butyldimethylsilyl)oxy)benzylidene)amino]-2-butene-1,4-dinitrile (**2**)

A mixture of 4-(*tert*-butyldimethylsilyloxy)benzaldehyde (2.26 g, 10.00 mmol), diaminomaleonitrile (**1**) (1.08 g, 10.00 mmol) and trifluoroacetic acid (3 drops) in MeOH (30 mL) was stirred for 10 min and a yellow solid precipitated out of solution. The solid was filtrated and washed with Et_2_O-hexane (1:1) (20 mL) to give imine **2** (2.83 g, 87% yield) as a yellow solid: mp 205 °C dec; R_f_ 0.41 (CH_2_Cl_2_); UV-Vis (CH_2_Cl_2_): λ_max_, nm (logε) 369 (4.36), 271 (3.96); ^1^H NMR (400 MHz, CDCl_3_) δ_H_ 8.37 (s, 1H, CH=Ph), 7.76–7.74 (d, ^3^*J* = 8.6 Hz, 2H, Ar), 6.93–6.91 (d, ^3^*J* = 8.6 Hz, 2H, Ar), 5.19 (bs, 2H, NH_2_), 1.00 (s, 9H, CH_3_), 0.26 (s, 6H, CH_3_); ^13^C NMR (100 MHz, CDCl_3_) δ_C_ 160.0, 158.6, 131.1.3, 128.3, 123.7, 120.7, 113.8, 112.6, 108.7, 25.6, 18.3, −4.3; HRMS *m*/*z* found: 327.1633 [M+H]^+^, 349.1454 [M+Na]^+^, 365.1196 [M+K]^+^ requires C_17_H_23_N_4_OSi 327.1641 [M+H]^+^, C_17_H_22_N_4_OSiNa 349.1461 [M+Na]^+^, C_17_H_22_N_4_OSiK 365.1199 [M+K]^+^.

2-amino-3-{[4-(*tert*-butyldimethylsilyl)oxy]benzylamino}-2-butene-1,4-dinitrile (**3**)

NaBH_4_ (228 mg, 6.00 mmol) was slowly added to a rapidly stirred suspension of imine **2** (750 mg, 4.00 mmol) in MeOH (25 mL). After the addition was complete, the solution was stirred for 30 min and poured into ice-H_2_O (200 mL). Solid was formed, filtrated and washed with H_2_O (50 mL) to give derivative **3** (984 mg, 75% yield) as a yellowish solid: mp 134–135 °C, R_f_ 0.56 (CH_2_Cl_2_); UV-Vis (CH_2_Cl_2_): λ_max_, nm (logε) 306 (3.83) ^1^H NMR (400 MHz, CDCl_3_) δ_H_ 8.31 (s, 1H, NH-), 7.77–7.75 (d, ^3^*J* = 8.6 Hz 2H, Ar), 6.92–6.90 (d, ^3^*J* = 8.6, 2H, Ar) 4.82 (s, 2H, NH_2_), 1.01 (s, 9H, CH_3_), 0.25 (s, 6H, CH_3_); ^13^C NMR (100 MHz, CDCl_3_) δ_C_ 159.4, 156.9, 130.6, 129.2, 128.9, 128.6, 127.2, 120.8, 120.6, 120.5, 112.8, 111.7, 110.7, 108.6, 49.9, 25.7, 18.3, −4.3; HRMS *m*/*z* found: 329.1789 [M+H]^+^, 351.1612 [M+Na]^+^, 367.1348 [M+K]^+^ requires C_17_H_25_N_4_OSi 329.11797 [M+H]^+^, C_17_H_24_N_4_OSiNa 351.1617 [M+Na]^+^, C_17_H_22_N_4_OSiK 367.1356 [M+K]^+^.

2-{[4-(*tert*-butyldimethylsilyl)oxy]benzylamino}-3-(2,5-dimethyl-1*H*-pyrrolyl)-butene-1,4-dinitrile (**4**)

MAOS synthesis—**3** (822 mg, 2.50 mmol), heksan-2,5-dione (330 µL, 2.75 mmol) oxalic acid (100 mg) and methanol (2 mL), were placed in a glass tube (G10) and sealed with silicon septum. The tube was placed in the microwave reactor and subjected to MAOS reaction (heat as fast as possible to 120 °C, hold for 10 min and cool to 55 °C). Flash column chromatography (dichloromethane) led to a mixture of *cis* and *trans* isomers of 4 (964 mg, 95% yield) as a yellow oil, R_f_ 0.7 (CH_2_Cl_2_); UV-Vis (CH_2_Cl_2_): λ_max_, nm (logε) 308 (3.89), 293 (3.93), 282 (3.94); ^1^H NMR (400 MHz, CDCl_3_) δ_H_ 9.69 (s, 1H, NH), 7.61–7.58 (d, ^3^*J* = 8.7 Hz, 1H, Ar), 6.88–6.85 (d, ^3^*J* = 8.6 Hz, 2H, Ar), 6.76–6.74 (d, ^3^*J* = 8.6 Hz, 1H, Ar), 6.64–6.62 (d, ^3^*J* = 8.6 Hz, 2H, Ar), 5.69 (s, 2H, 3,4-H-pyrrole), 4.22–4.20 (d, 2H, CH_2_-Ph), 1.85 (s, 6H, 2,5-CH_3_-pyrrole), 0.80 (s, 4.6 H, CH_3_), 0.78 (s, 9H, CH_3_), 0.06 (s, 2.61 H, CH_3_) 4.82 (s, 2H), 1.01 (s, 9H), 0.25 (s, 6H), 0.00 (s, 6H); ^13^C NMR (100 MHz, CDCl_3_) δ_C_ 190.9, 156.1, 132.5, 131.9, 128.9, 128.8, 128.3, 115.2, 110.8, 108.7, 108.6, 91.9, 49.8, 25.6, 18.2, 11.9, −4.4; HRMS *m*/*z* found: 429.2094 [M+Na]^+^, 445.1826 [M+K]^+^ requires C_23_H_30_N_4_OSiNa 429.2087 [M+Na]^+^, C_23_H_30_N_4_OSiK 445.1826 [M+K]^+^.

2-{Methyl[4-(*tert*-butyldimethylsilyl)oxy]benzylamino}-3-(2,5-dimethyl-1*H*-pyrrolyl)-butene-1,4-dinitrile (**5**)

MAOS synthesis—**4** (812 mg, 2.00 mmol), methyl iodide (245 µL, 4.00 mmol) cesium carbonate (651 mg, 2.00 mmol) and THF (2 mL), were placed in a glass tube (G10) and sealed with silicon septum. The tube was situated in the Monowave 400 reactor and subjected to MAOS reaction following the conditions: heat as fast as possible to 120 °C, hold for 10 min and cool to 55 °C. Flash column chromatography (dichloromethane) led to a mixture of *cis* and *trans* isomers of **5** (756 mg, 90% yield) as a yellowish oil: R_f_ 0.62 (CH_2_Cl_2_); UV-Vis (CH_2_Cl_2_): λ_max_, nm (logε) 320 (3.95), 284 (3.78); ^1^H NMR (400 MHz, CDCl_3_) δ_H_ 6.94–6.91 (d, ^3^*J* = 8.6 Hz, 2H, Ar), 6.88–6.86 (d, ^3^*J* = 8.6 Hz, 2H, Ar), 6.67–6.65 (d, ^3^*J* = 8.6 Hz, 2H, Ar), 6.63–6.61 (d, ^3^*J* = 8.6 Hz, 2H, Ar), 5.66 (s, 2H, 3,4-H-pyrrole), 5.55 (s, 2H, 3,4-H-pyrrole), 4.45 (s, 2H, CH_2_-Ph), 4.17 (s, 2H, CH_2_-Ph), 3.08 (s, 3H, CH_3_-N), 2.16 (s, 3H, CH_3_-N), 1.97 (s, 6H, 2,5-CH_3_-pyrrole), 1.84 (s, 6H, 2,5-CH_3_-pyrrole), 0.77 (s, 9H, CH_3_), 0.77 (s, 9H, CH_3_), 0.00 (s, 6H, CH_3_), −0.02 (s, 6H, CH_3_); ^13^C NMR (100 MHz, CDCl_3_) δ_C_ 156.3, 135.7, 127.1 126.9, 120.9, 120.8 117.0 116.4, 112.9, 110.9 10.7 107.6, 90.5, 88.8, 59.7, 59.0 39.8 59.0 39.8 36.6 25.7, 25.6 18.2 12.4, 12.3, −4,4; HRMS *m*/*z* found: 443.2241 [M+Na]^+^, 459.1939 [M+K]^+^ requires C_24_H_32_N_4_OSiNa 443.1978 [M+Na]^+^, C_24_H_32_N_4_OSiK 459.1982 [M+K]^+^.

[2,7,12,17-Tetrakis{methyl[4-(*tert*-butyldimethylsilyl)oxy]benzylamino}-3,8,13,18-tetrakis(2,5-dimethyl-1*H*-pyrrol-1-yl)porphyrazinato]magnesium(II) (**6**)

MAOS synthesis—**5** (420 mg, 1.00 mmol), magnesium(II) *tert*-butoxide (85 mg, 0.50 mmol) and 1-butanol (2 mL), were placed in a glass tube (G10) and sealed with silicon septum. The tube was placed in the microwave reactor and subjected to MAOS reaction (heat as fast as possible to 180 °C, hold for 10 min and cool to 70 °C). Flash column chromatography (dichloromethane) led to **6** (247 mg, 58% yield) as a dark green film: mp ≥ 300 °C, R_f_ 0.41 (CH_2_Cl_2_:CH_3_OH 50:1); UV-Vis (CH_2_Cl_2_): λ_max_, nm (logε) 732 (4.56), 653 (4.21), 451 (4.01), 349 (4.71); ^1^H NMR (400 MHz, pyridine-*d*_5_) δ_H_ 7.35–7.33 (d, ^3^*J* = 8.6 Hz, 8H, Ar), 6.87–6.84 (d, ^3^*J* = 8.6 Hz, 8H, Ar), 6.18 (s, 8H, 3,4-H-pyrrole), 6.00 (s, 8H, -CH_2_-Ph), 3.21 (s, 12H, CH_3_-N), 2.14 (s, 26H, 2,5-CH_3_-pyrrole), 0.91 (s, 36H, CH_3_), 0.13 (s, 24H, CH_3_); ^13^C NMR (100 MHz pyridine-*d*_5_) δ_C_ 154.9, 154.3, 151.2, 148.0, 134.8, 132.4, 131.9, 129.6, 123.8, 122.7, 120.0, 112.1, 105.9, 57.0, 38.9, 25.5, 18.1, 13.3, −4.7; HRMS *m*/*z* found: 1705.9307 [M+H]^+^ requires C_96_H_129_MgN_16_O_4_Si_4_ 1705.9310 [M+H]^+^.

2,7,12,17-Tetrakis[methyl(4-hydroxy)benzylamino]-3,8,13,18-tetrakis(2,5-dimethyl-1*H*-pyrrol-1-yl) porphyrazinato]magnesium(II) (**7**)

A solution of tetrabutylammonium fluoride (80 mg, 0.30 mmol) in THF (2 mL) was added to a solution of **6** (100 mg, 0.06 mmol) in THF (5 mL) at 0 °C. The reaction was allowed to stir for 120 min before quenching with water. The aqueous layer was extracted with DCM (3 × 30 mL) and the combined organic layers were dried over magnesium sulfate, filtered and concentrated. Flash column chromatography (dichloromethane) led to **7** (65 mg, 87% yield) as a dark green film: mp ≥ 300 °C, R_f_ 0.43 (CH_2_Cl_2_:CH_3_OH 10:1); UV-Vis (CH_2_Cl_2_): λ_max_, nm (logε) 716 (4.31), 644 (4.16), 344 (4.76), 278 (4.81); ^1^H NMR (400 MHz, pyridine-*d*_5_) δ_H_ 11.41 (bs, 4H, OH), 7.35–7.33 (d, ^3^*J* = 8.6 Hz, 8H, Ar), 7.06–7.04 (d, ^3^*J* = 8.6 Hz, 8H, Ar), 6.19 (s, 8H, 3,4-H-pyrrole), 5.97 (bs, 8H, -CH_2_-Ph), 3.22–3.19 (m, 12H, CH_3_-N), 2.17 (bs, 24H, 2,5-CH_3_-pyrrole); ^13^C NMR (100 MHz pyridine-*d*_5_) δ_C_ 157.9, 132.1, 131.9, 129.9, 115.8, 105.9, 98.3, 81.3, 38.6, 13.3; HRMS *m*/*z* found: 1249.5848 [M+H]^+^ requires, C_72_H_73_MgN_16_O_4_. 1249.5851 [M+H]^+^.

### 3.3. Electrochemical Measurements

Electrochemical experiments were conducted on the Metrohm Autolab PGSTAT128N potentiostat. Data acquisition was driven by Metrohm Nova 2.1.4 software. Measurements were carried out on a glassy carbon (GC) working electrode (3 mm, area = 0.02 cm^2^), with Ag wire as a pseudo-reference electrode, and Pt wire as a counter electrode in a heart-shaped glass cell (volume 10 mL). Before performing the electrochemical experiments, the GC electrode was polished with an aqueous 50 nm Al_2_O_3_ slurry (Sigma-Aldrich, St. Louis, MO, USA) on a diamond polishing cloth (BASi), followed by subsequent washing in an ultrasonic bath with water/acetone mixture (1:1, *v*/*v*) for 12 min in order to remove any impurities. Ferrocene (Sigma-Aldrich) was used as an internal standard. Prior to each measurement, a solution of dichloromethane (DCM) with a supporting electrolyte (0.1 M tetrabutylammonium perchlorate, TBAP, Sigma-Aldrich) was deoxygenated by purging nitrogen gas for 20 min. All electrochemical experiments were carried out at room temperature.

### 3.4. Spectroelectrochemical Measurements

Spectroelectrochemical experiments were performed on the Ocean Optics USB 2000+XR1-ES diode (Orlando, FL, USA) array spectrophotometer and Metrohm Autolab PGSTAT128N potentiostat (Barendrecht, The Nederlands). A quartz cuvette (2 mm optical path length) was used along with a Pt gauze working electrode, with an Ag/AgCl as a reference, and a platinum wire as an auxiliary electrode. The spectra were recorded in deoxygenated DCM/0.1 M TBAP solution in the range of 200–1000 nm within 2 min (every 10 s) during application of proper overpotential based on DPV voltammograms.

### 3.5. Fluorescence Measurements

A Jasco 6200 spectrofluorometer (Tokyo, Japan) with Spectra Manager version 1.55 was used for the recording of the emission spectra. Measurements were made at ambient temperature, using 1 cm-path-length quartz cuvettes. The diluted solutions (absorbance was kept below 0.1) of porphyrazines and unsubstituted zinc(II) phthalocyanine used as a reference were excited at λ = 630 nm. The fluorescence quantum yields Φ_F_ were calculated according to the following equation:

$$\Phi_{Fs}=\Phi_{Fr}\times \frac{F_{s}}{F_{r}}\times \frac{{1-10}^{{-A}_{r}}}{{1-10}^{{-A}_{s}}}$$

where F is the area under the emission curve for the sample (F_s_) and the reference (F_r_), A is the absorbance at the excitation wavelength for the sample (A_s_) and the reference (A_r_), and Φ_Fr_ is the fluorescence quantum yield of the reference (Φ_Fr_ = 0.17 and 0.20 in DMF and DMSO, respectively) [51,52].

### 3.6. Singlet Oxygen-Generation Quantum Yield Measurements

The assessment of singlet oxygen-generation quantum yield for the porphyrazine was performed in DMF and DMSO solution under air conditions at ambient temperature. 1,3-Diphenylisobenzofuran (DPBF, Sigma-Aldrich) was used as a chemical quencher of singlet oxygen. DPBF and Pz mixture was irradiated with the wavelength light adjusted to the maximum absorbance of the macrocycle. Unsubstituted zinc(II) phthalocyanine with known singlet oxygen quantum yields (0.56 in DMF, 0.67 in DMSO) was used as a reference [51,52]. The change in absorbance was monitored at 417 nm under light irradiation. The rate at which the quencher degrades is related to the generation of singlet oxygen.

### 3.7. Microtox Acute Toxicity Assessment

The Microtox acute toxicity test was performed with the use of a Microtox M500 photometer (Modern Water, London, UK) and the data were collected using Modern Water Microtox Omni 4.2. For the measurements, an 81.9% screening test procedure was applied. Due to the insolubility of the tested porphyrazines in water, an addition of dimethylsulfoxide was used as the solubilizator, the content of which did not exceed 1%.

### 3.8. Antimicrobial Assay

Bacteria and growth conditions.

The microorganism used in the study was a clinical strain of *Staphylococcus aureus* with methicillin resistance mechanism (MRSA). Bacterial strain was stored in Microbank cryogenic vials (ProLabDiagnostics, Richmond Hill, ON, Canada) at −70 °C ± 10 °C. The bacteria were cultured in Brain-heart infusion broth (BHI, OXOID, GB) at 36 °C ± 1 °C for 18 h.

#### 3.8.1. Determination of the Dark Toxicity

The microorganisms were harvested by centrifugation and re-suspended in 0.9% NaCl solution to a final concentration of about 10^7^ colony-forming units (CFUs)/mL. Aliquots (100 µL) of a standardized microbial suspension were placed in the wells of microtitre plates, and a solution of tested compounds was added to give final concentrations of 10^−4^ M. Control wells contained the microbial suspension in 0.9% NaCl without the tested compounds. Control tests were also performed with 2% solution of methanol in water, which was used as a solvent for the tested compounds. All the samples were incubated in the dark for 60 min, and then the number of viable microbial cells in the samples was determined with the plate count method using the Tryptic soy agar (TSA, OXOID, UK). The log reduction in viable microorganisms was calculated based on the number of colony-forming units (CFUs) evaluated.

#### 3.8.2. Photodynamic Inactivation of Microbial Cells

The samples were prepared as described above. After incubation in the dark, the samples were irradiated at room temperature with high-power LED MultiChip Emitters (60 high-efficiency AlGaAs diode chips, Roithner LaserTechnik GmbH, Vienna, Austria) with a maximum wavelength of 730 ± 15 nm at a fixed total light dose 3 mW/cm^2^. Viable microbial cells were measured similarly to dark toxicity to evaluate the photodynamic inactivation. The number of CFUs was calculated, and the reduction in viable cells was determined in relation to the control samples containing microbes and saline alone (Table S1).

## 4. Conclusions

The study focused on two magnesium(II) derivatives of porphyrazines: compound **6** (with silyl protected hydroxyl groups) and its deprotected form—compound **7**. The synthesis was carried out in several stages, combining classical methods and the MAOS technique. The obtained compounds were characterized using such spectral techniques as UV-Vis, MS ES, ^1^H and ^13^C NMR. By using cyclic (CV) and differential pulse voltammetries (DPV), the redox properties of both compounds were studied in DCM solution with TBAP electrolyte. In the analyzed potential window, the electrochemical activity was observed by means of four redox processes—one reduction and three oxidation processes. Due to the lack of electrochemical activity of the Mg(II) ion, these processes were attributed to the macrocyclic ligand. In porphyrazine **7**, due to its poor solubility in dichloromethane, the redox signals were less pronounced. Particularly noticeable was the shift of the third oxidation process (Oxd_3_) in **7** towards more positive potentials, which is associated with the presence of free hydroxyl groups susceptible to oxidation. Spectroelectrochemical studies revealed the formation of cationic forms of macrocyclic ligands at 1.2 V in both cases. Characteristic Q (718–731 nm) and Soret (~350 nm) absorption bands were observed in both porphyrazines. The obedience to the Lambert–Beer law suggested the lack of aggregation. Derivative **6** showed slightly higher fluorescence quantum yields, but they were low for both compounds (<0.004). No emission from the S_2_ state was observed, in contrast to other porphyrazines with pyrrole moieties. The quantum yield of singlet oxygen generation (Φ_Δ_) was measured. Compound **6** showed higher efficiency in generating singlet oxygen than **7** (Φ_Δ_ up to 0.06 vs. 0.02, respectively), although this was still lower than some of the previously described pyrrole porphyrazines (even up to 0.27).

In the Microtox test, compound **6** proved to be more toxic than **7**. The threshold of the 20% reduction in luminescence (indicating toxicity) was reached for **6** at a concentration of 2 × 10^−6^ M, while for **7** it was only above 10^−5^ M.

A study of photodynamic inactivation of MRSA was performed. The results showed clear differences between the tested compounds: porphyrazine **7** showed strong bactericidal activity under light irradiation, reducing the number of bacteria by more than 5.6 log. In contrast, porphyrazine **6** did not show antimicrobial activity, neither in the dark nor under the influence of light. The action of the compounds was confirmed as photodynamic, because no bactericidal effects were observed without light. However, the mechanism of the photodynamic action is not fully revealed, due to the low singlet oxygen-generation quantum yields for both Pzs. In summary, compound **7** showed the greatest application potential as a photosensitizer, combining good photodynamic activity with low toxicity. The results suggest its suitability for further studies as a potential photosensitizer in photodynamic antimicrobial chemotherapy.

## Figures and Tables

**Table 1:** Electrochemical data (E_1/2_ [V] vs. Fc^+^/Fc) of the obtained magnesium(II) porphyrazines **6** and **7** based on DPV measurements.

Comp.	Red(Pz^−1^/Pz)	Oxd_1_(Pz/Pz^+1^)	Oxd_2_(Pz^+1^/Pz^+2^)	Oxd_3_(Pz^+2^/Pz^+3^)
**6**	−1.96	−0.32	0.17	0.34
**7**	−1.94	−0.19	0.03	0.70

**Table 2:** Summary of photophysical properties of Pzs **6** and **7** in solution.

Compound	Pz 6		Pz 7	
Solvent	DMF	DMSO	DMF	DMSO
Φ_Δ_	0.05	0.06	0.02	0.02
Φ_F_	0.004	0.002	<0.001	<0.001
absorption maxima λ_max_ [nm], (molar absorption coefficients—log ε [M^−1^·cm^−1^])	725 (4.75); 351 (4.89)	731 (4.65); 351 (4.81)	718 (4.10); 350 (4.39)	723 (4.11); 350 (4.41)
emission maximum λ_F_ [nm]	742	751	744	750
Stokes shift [cm^−1^]	316	364	487	498

**Table 3:** The bactericidal activity of studied porphyrazines against MRSA.

Compound		Pz 6	Pz 7	Light Fluence [J/cm^2^]
Conditions	Irradiation Time	Log Reduction in Bacterial Growth		
Light	60 min	−0.01 ± 0.09	>5.68 ± 0.05	10.8
	90 min	0.01 ± 0.05	>5.66 ± 0.05	16.2
Dark	60 min	−0.01 ± 0.07	0.05 ± 0.03	10.8
	90 min	0.07 ± 0.04	0.09 ± 0.02	16.2

## Data Availability

All additional data are available in the Supplementary Materials File.

## Supplementary Materials

The following supporting information can be downloaded at https://www.mdpi.com/article/10.3390/molecules30153069/s1, Figure S1. UV-Vis spectra of Pz **6** in DMF at different concentrations (left), and correlation between concentration and absorbance at the wavelength corresponding to the absorption peaks (right). Figure S2. UV-Vis spectra of Pz **6** in DMSO at different concentrations (left), and correlation between concentration and absorbance at the wavelength corresponding to the absorption peaks (right). Figure S3. UV-Vis spectra of Pz **7** in DMF at different concentrations (left), and correlation between concentration and absorbance at the wavelength corresponding to the absorption peaks (right). Figure S4. UV-Vis spectra of Pz **7** in DMSO at different concentrations (left), and correlation between concentration and absorbance at the wavelength corresponding to the absorption peaks (right). Figure S5. Absorption, emission and excitation spectra of Pz **6** in DMF. Figure S6. Absorption, emission and excitation spectra of Pz **6** in DMSO. Figure S7. Absorption, emission and excitation spectra of Pz **7** in DMF. Figure S8. Absorption, emission and excitation spectra of Pz **7** in DMSO. Table S1. The bactericidal activity of 2% solution of methanol in water, used to dissolve porphyrazines, against MRSA; Figure S9. ^1^H NMR, 400 MHz, CDCl_3_ of **2**; Figure S10. ^13^C NMR 100 MHz, CDCl_3_ of **2**; Figure S11. ^1^H NMR, 400 MHz, CDCl_3_ of **3**; Figure S12. ^13^C NMR 100 MHz, CDCl_3_ of **3**; Figure S13. ^1^H NMR, 400 MHz, CDCl_3_ of **4**; Figure S14. ^13^C NMR 100 MHz, CDCl_3_ of **4**; Figure S15. ^1^H NMR, 400 MHz, CDCl_3_ of **5**; Figure S16. ^13^C NMR 100 MHz, CDCl_3_ of **5**; Figure S17. ^1^H NMR, 400 MHz, pyridine-*d*_5_ of **6**; Figure S18. ^13^C NMR 100 MHz, pyridine-*d*_5_ of **6**; Figure S19. ^1^H NMR, 400 MHz, pyridine-*d*_5_ of **7**; Figure S20. ^13^C NMR 100 MHz, pyridine-*d*_5_ of **7**; Figure S21. HMRS of **2**; Figure S22. HRMS of **3**; Figure S23. HRMS of **4**; Figure S24. HRMS of **5**; Figure S25. HRMS of **6**; Figure S26. HRMS of **7**.
