# Supplementary material for: Novel Tetraphenolic Porphyrazine Capable of MRSA Photoeradication
Source: Molecules. 2025 Jul 22;30(15):3069. doi: 10.3390/molecules30153069 (PMC12348665; doi:10.3390/molecules30153069)
Supplement: Supplementary file 1 [file molecules-30-03069-s001.zip › molecules-3762903-supplementary.pdf]

Novel tetraphenolic porphyrazine capable of MRSA photoeradication

Wojciech Szczolko[a,*], Eunice Zuchowska[a], Tomasz Koczorowski[a], Michal Kryjewski[b], Jolanta Dlugaszewska[c], Dariusz T. Mlynarczyk[a]

[a] Chair and Department of Chemical Technology of Drugs, Poznan University of Medical Sciences, Rokietnicka 3, 60-806 Poznan, Poland

[b] Chair and Department of Inorganic and Analytical Chemistry, Poznan University of Medical Sciences, Rokietnicka 3, 60-806 Poznan, Poland

[c] Chair and Department of Genetics and Pharmaceutical Microbiology, Poznan University of Medical Sciences, Rokietnicka 3, 60-806 Poznan, Poland

Table of contents

1. Absorption and emission properties S2
2. Antibacterial activity of the medium used in experiments S6
3. NMR spectra of the compounds S7
4. HRMS spectra of the compounds S13

1. Absorption and emission properties

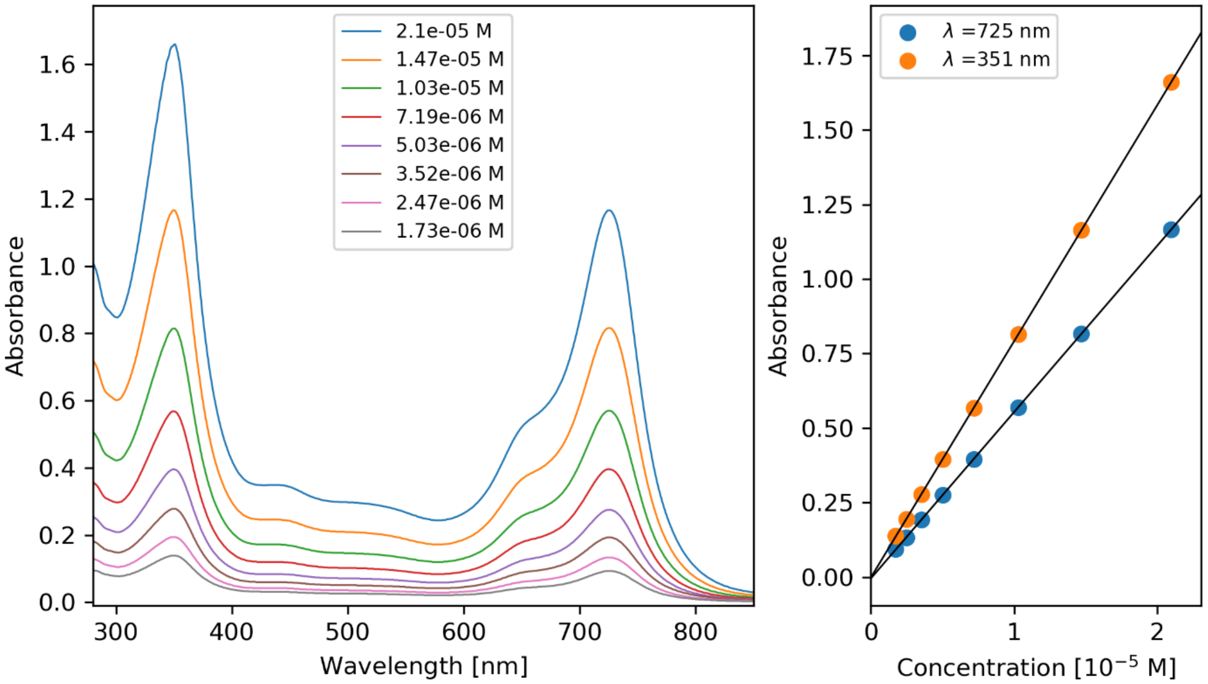

Figure S1 UV-Vis spectra of **Pz 6** in DMF at different concentrations (left), and correlation between concentration and absorbance at the wavelength corresponding to the absorption peaks (right).

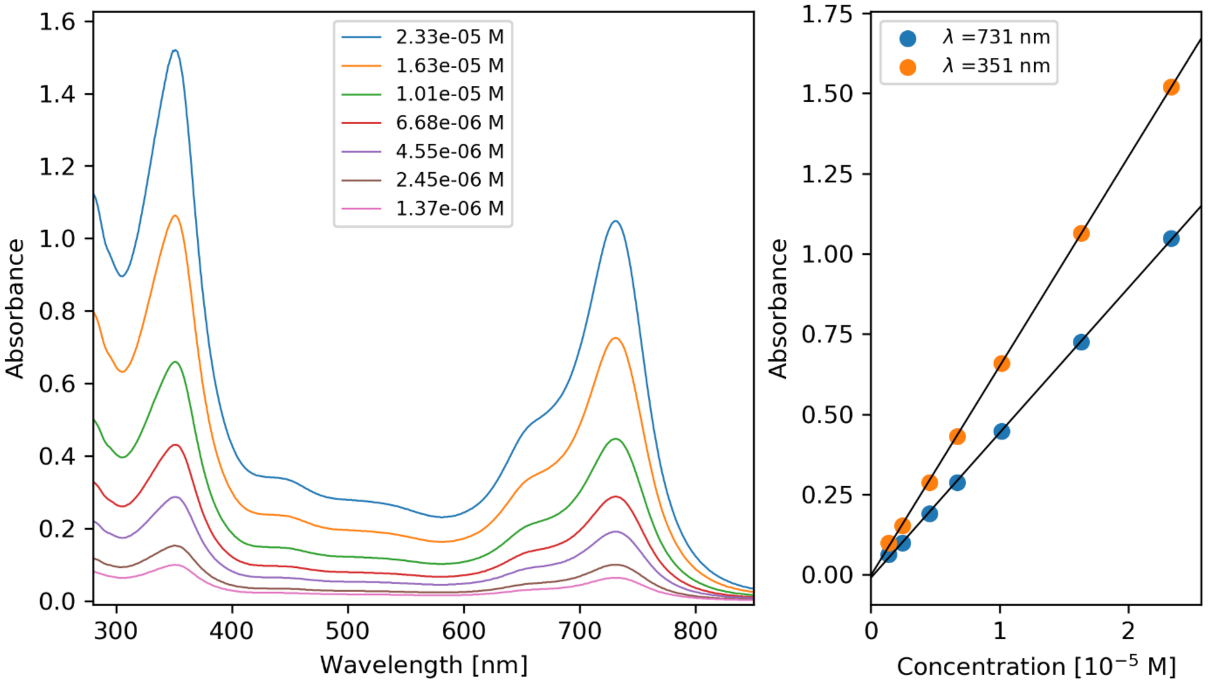

Figure S2 UV-Vis spectra of **Pz 6** in DMSO at different concentrations (left), and correlation between concentration and absorbance at the wavelength corresponding to the absorption peaks (right).

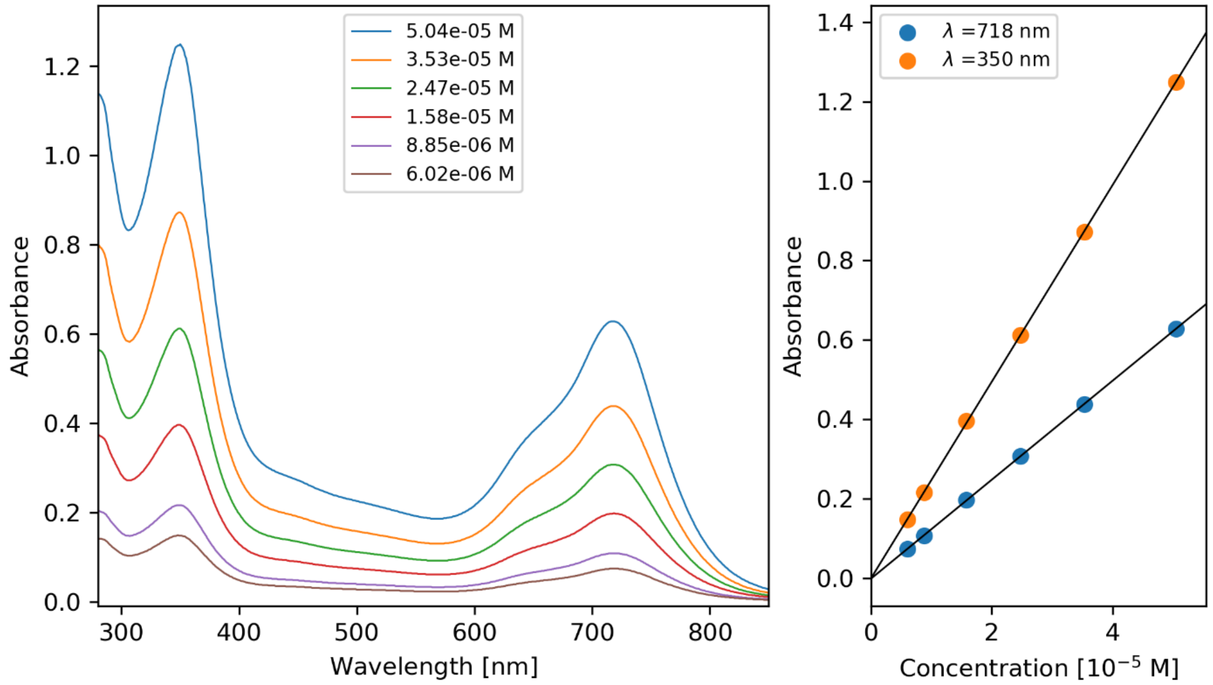

Figure S3 UV-Vis spectra of **Pz 7** in DMF at different concentrations (left), and correlation between concentration and absorbance at the wavelength corresponding to the absorption peaks (right).

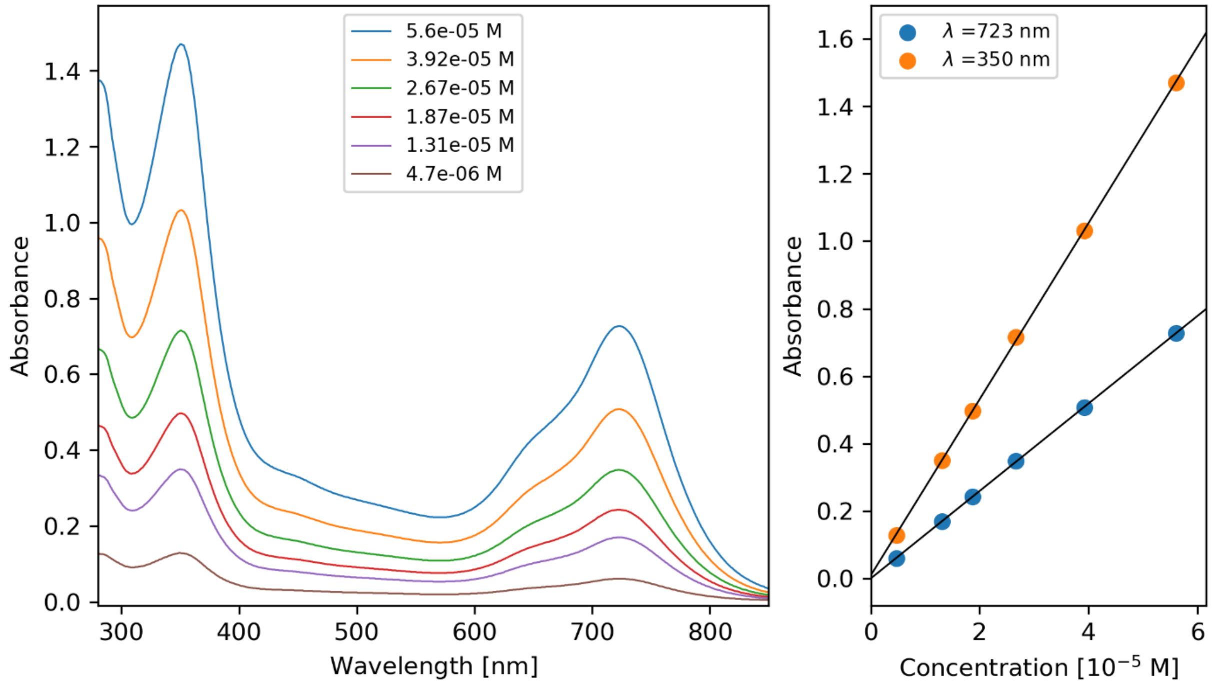

Figure S4 UV-Vis spectra of **Pz 7** in DMSO at different concentrations (left), and correlation between concentration and absorbance at the wavelength corresponding to the absorption peaks (right).

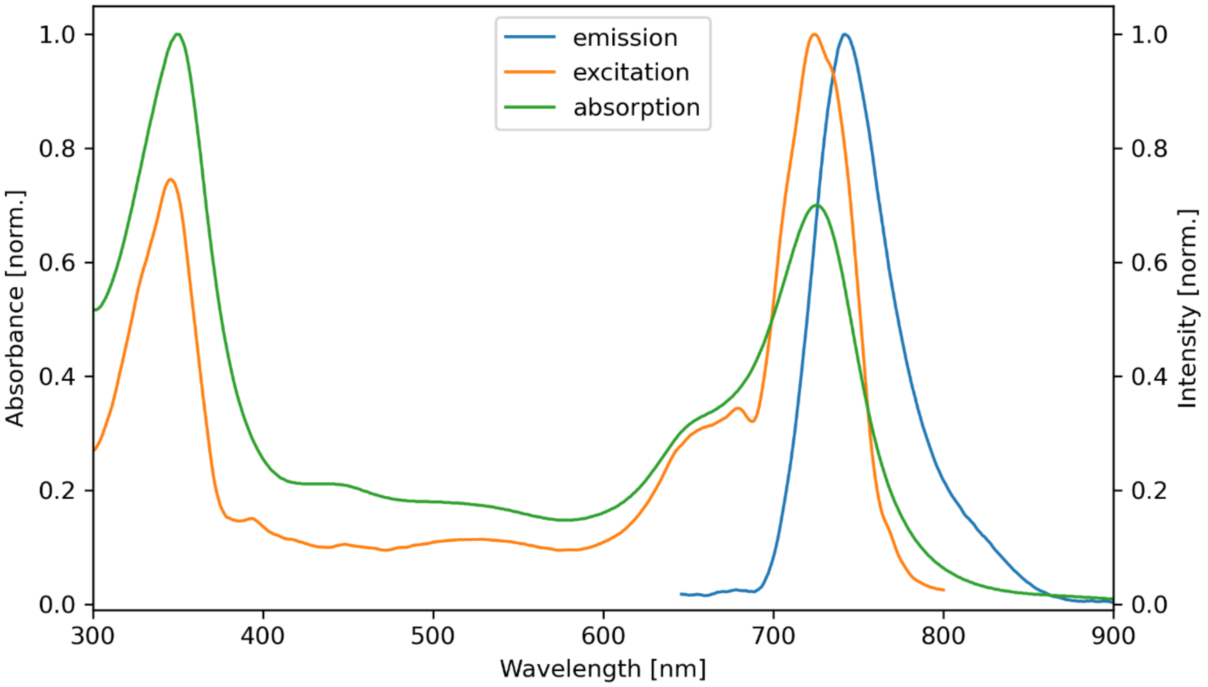

Figure S5. Absorption, emission and excitation spectra of **Pz 6** in DMF.

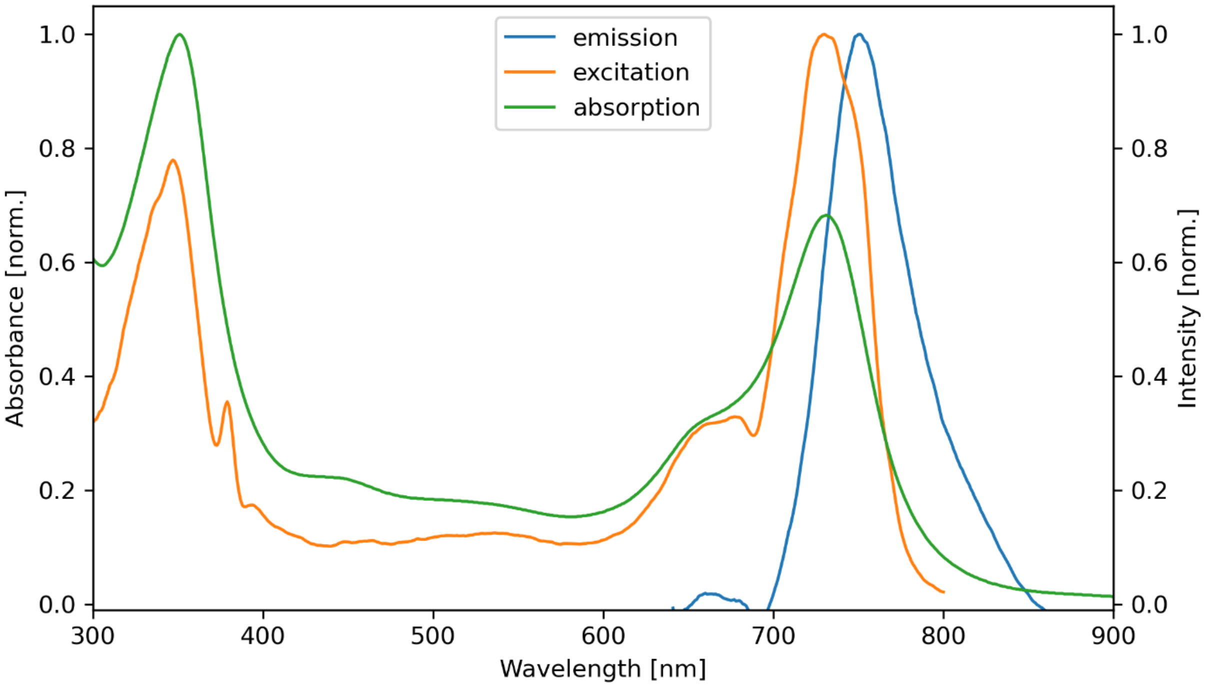

Figure S6. Absorption, emission and excitation spectra of **Pz 6** in DMSO.

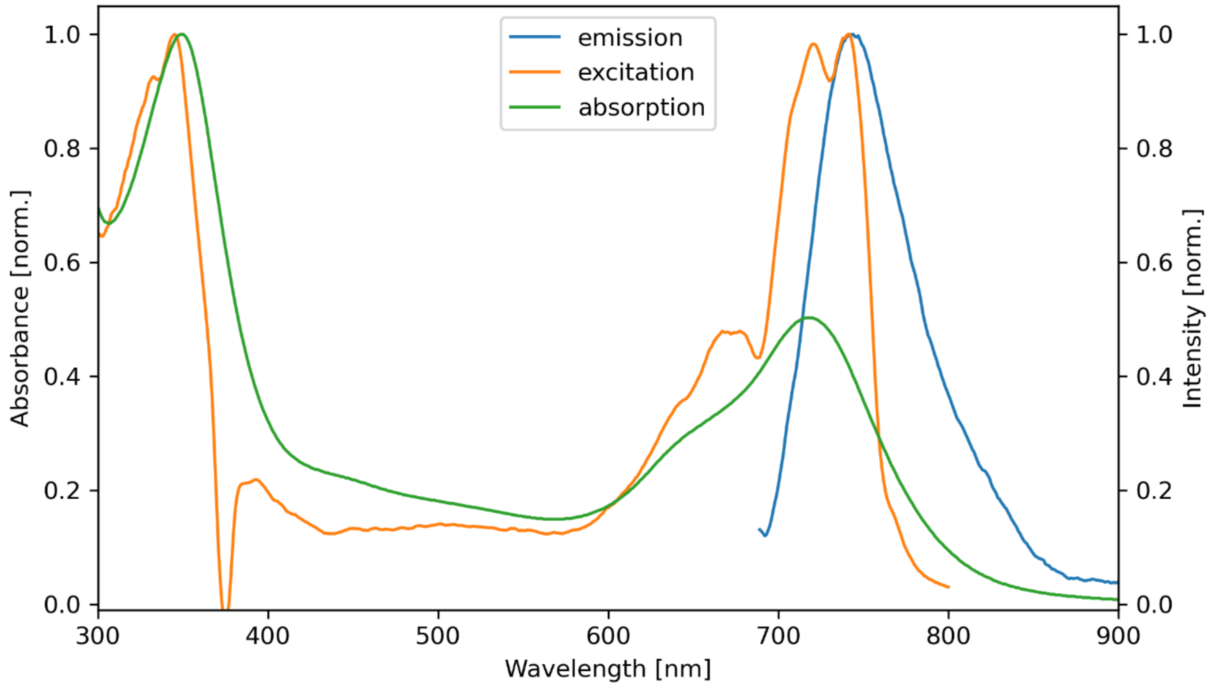

Figure S7. Absorption, emission and excitation spectra of **Pz 7** in DMF.

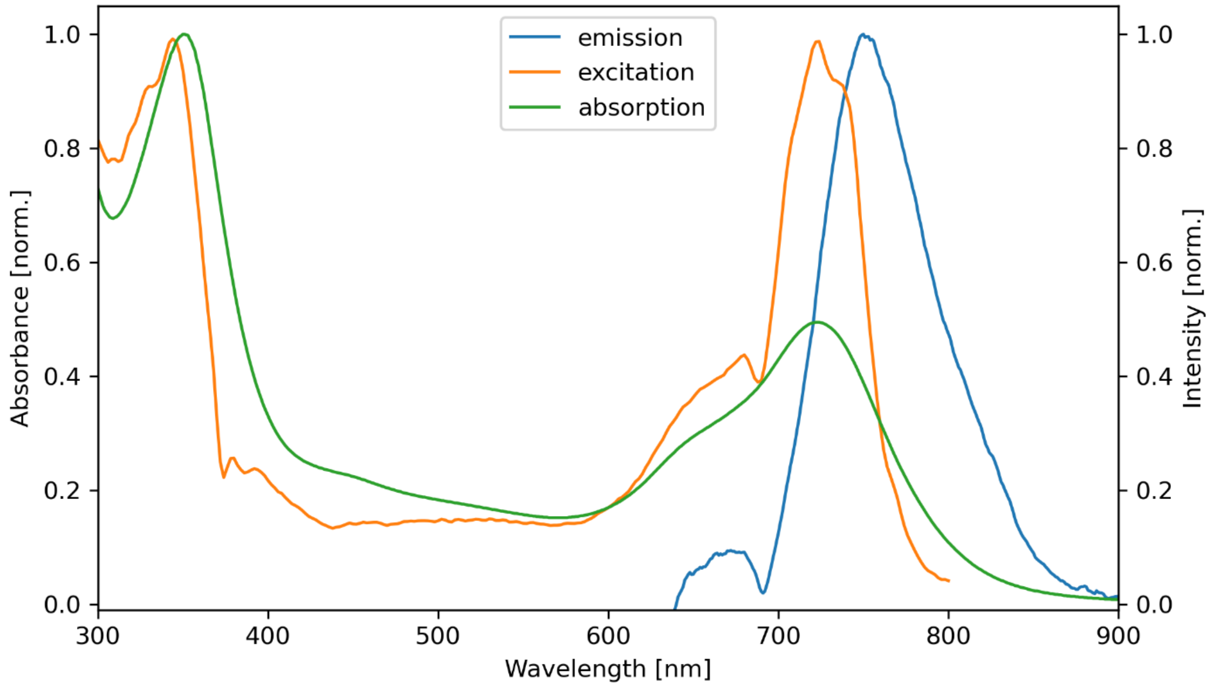

Figure S8. Absorption, emission and excitation spectra of **Pz 7** in DMSO.

2. Antibacterial activity of the medium used in experiments

Table S1. The bactericidal activity of 2% solution of methanol in water, used to dissolve porphyrazines, against MRSA

Compound		2% solution of methanol in water
Conditions	Irradiation time	log reduction in bacterial growth
Light	60 min	0.11 ± 0.06
	90 min	0.02 ± 0.04
Dark	60 min	0.01 ± 0.08
	90 min	0.02 ± 0.04

3. NMR spectra of the compounds

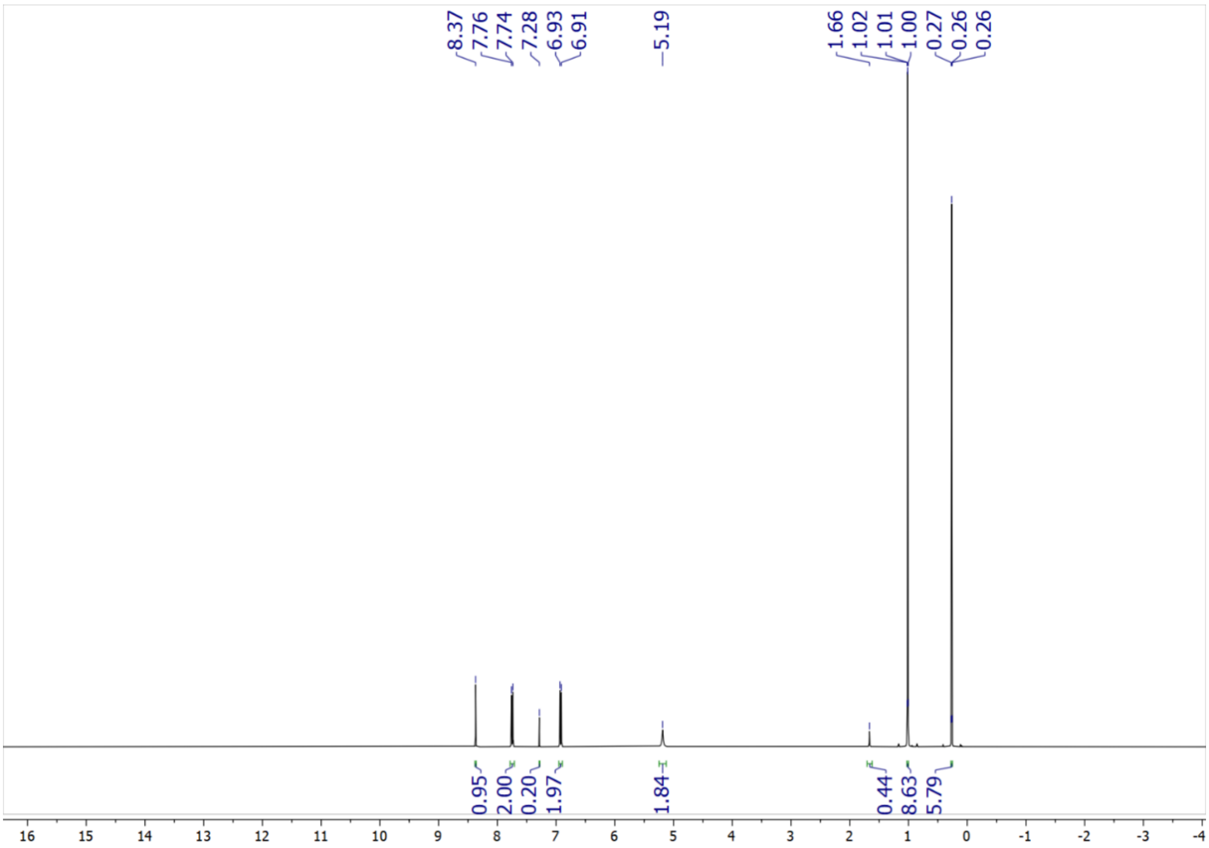

Figure S9. ^{1}H NMR, 400 MHz, $CDCl_3$ of **2**

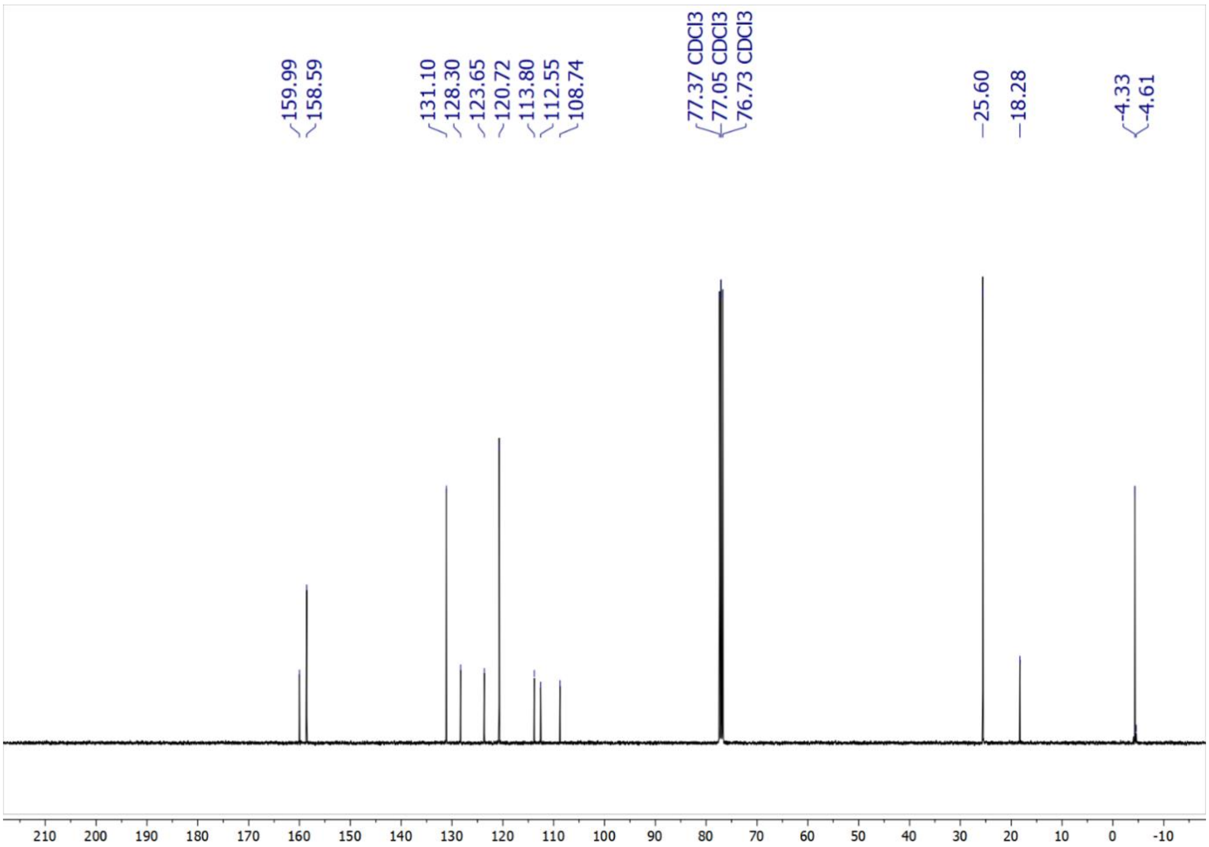

Figure S10. ^{13}C NMR 100 MHz, $CDCl_3$ of **2**

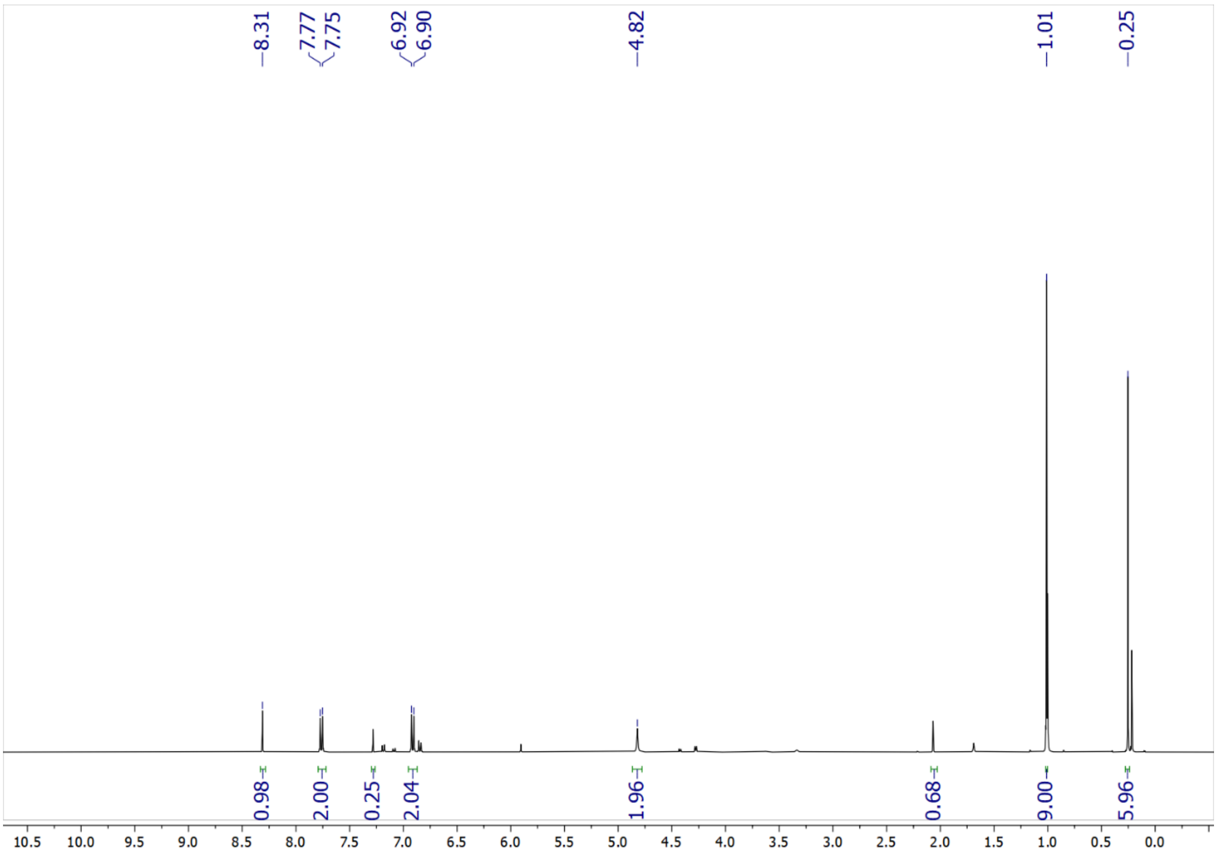

Figure S11. ^{1}H NMR, 400 MHz, $CDCl_3$ of **3**

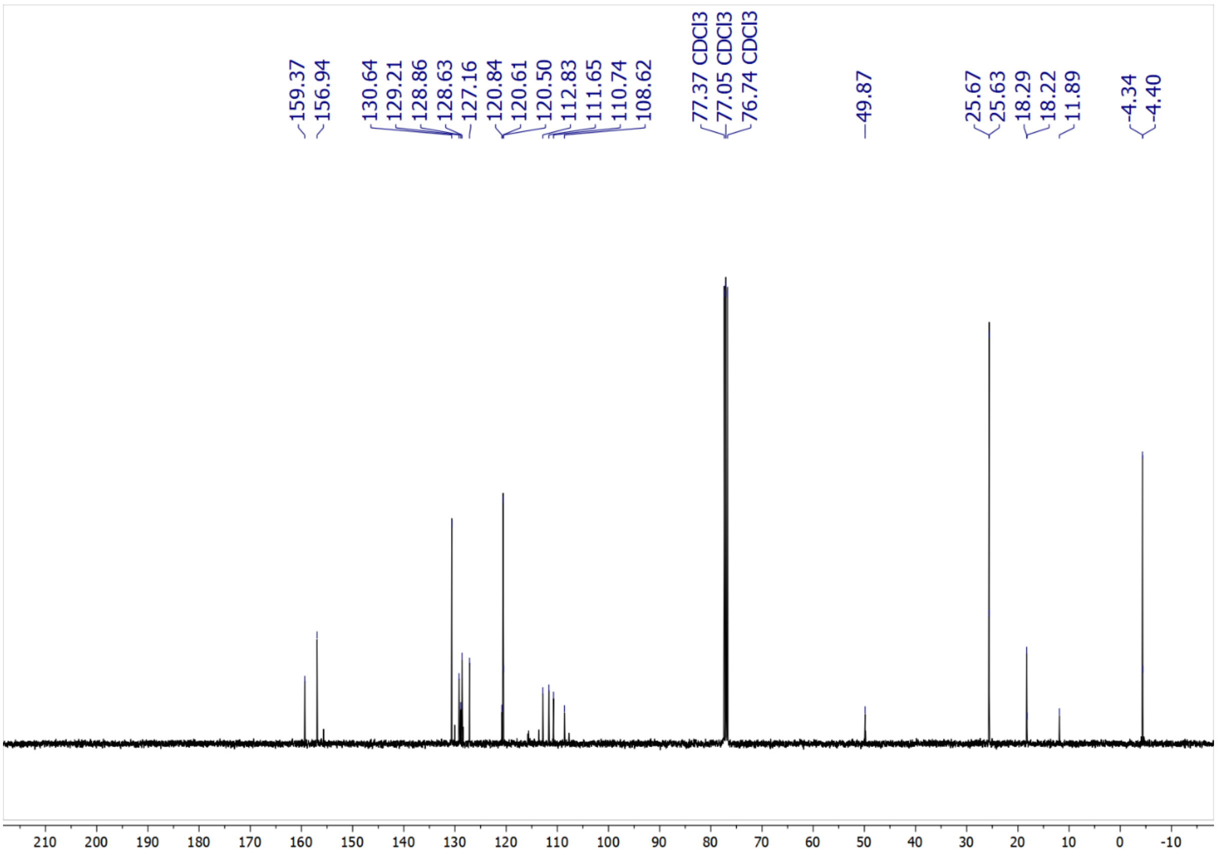

Figure S12. ^{13}C NMR 100 MHz, $CDCl_3$ of **3**

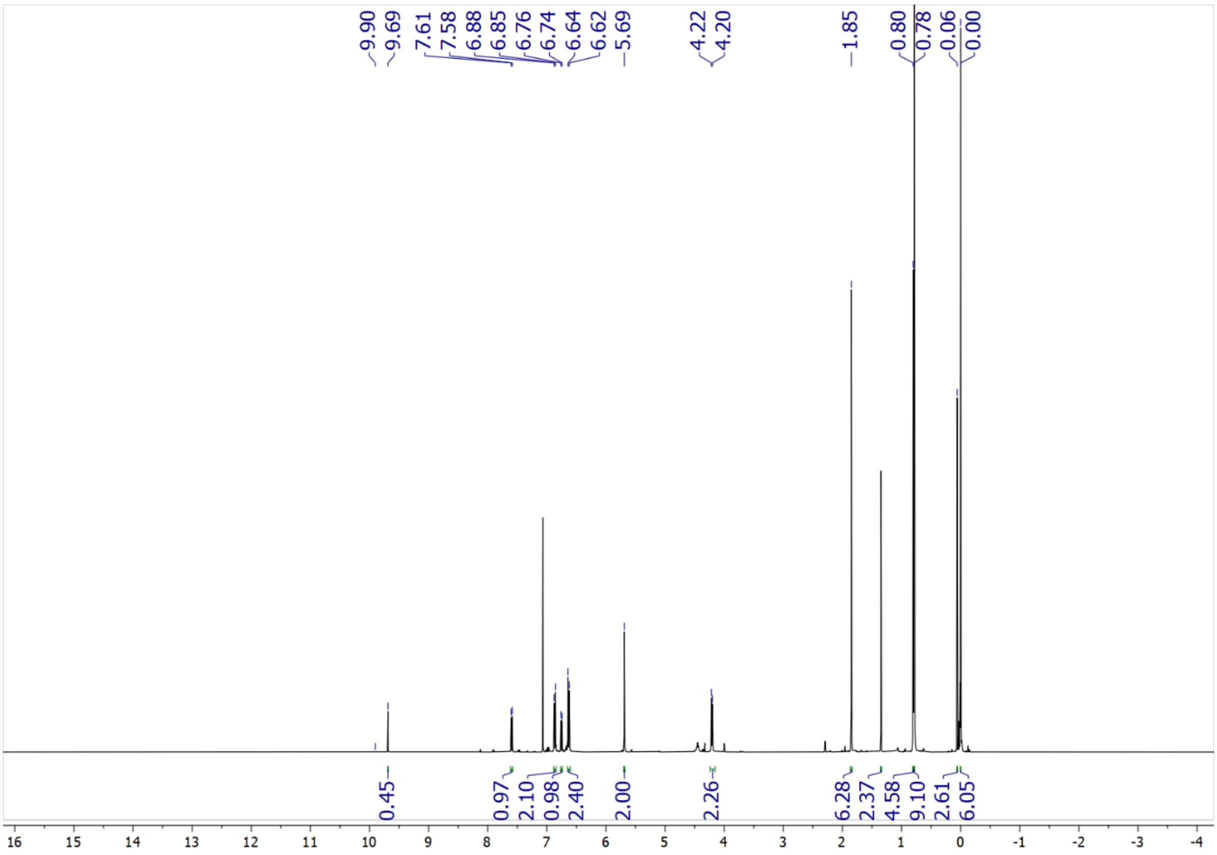

Figure S13. ^{1}H NMR, 400 MHz, $CDCl_3$ of **4**

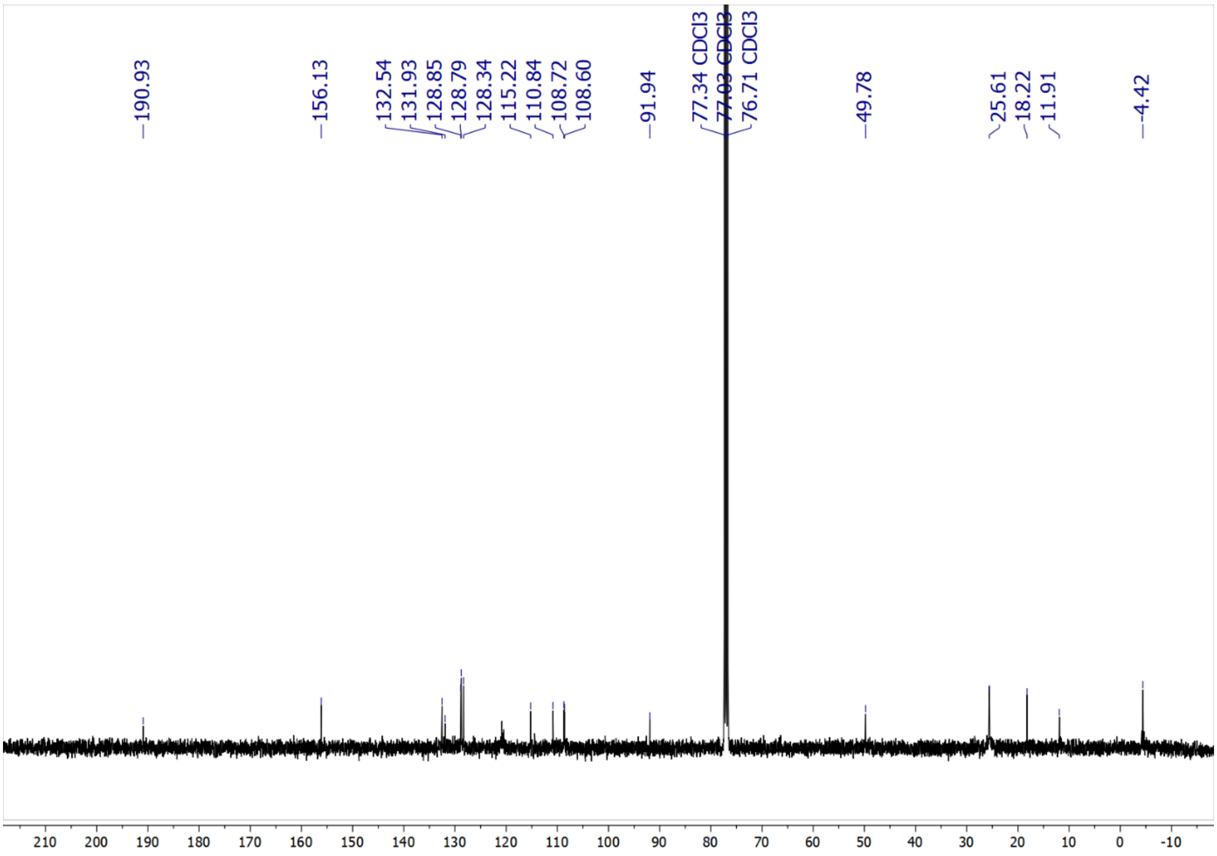

Figure S14. ^{13}C NMR 100 MHz, $CDCl_3$ of **4**

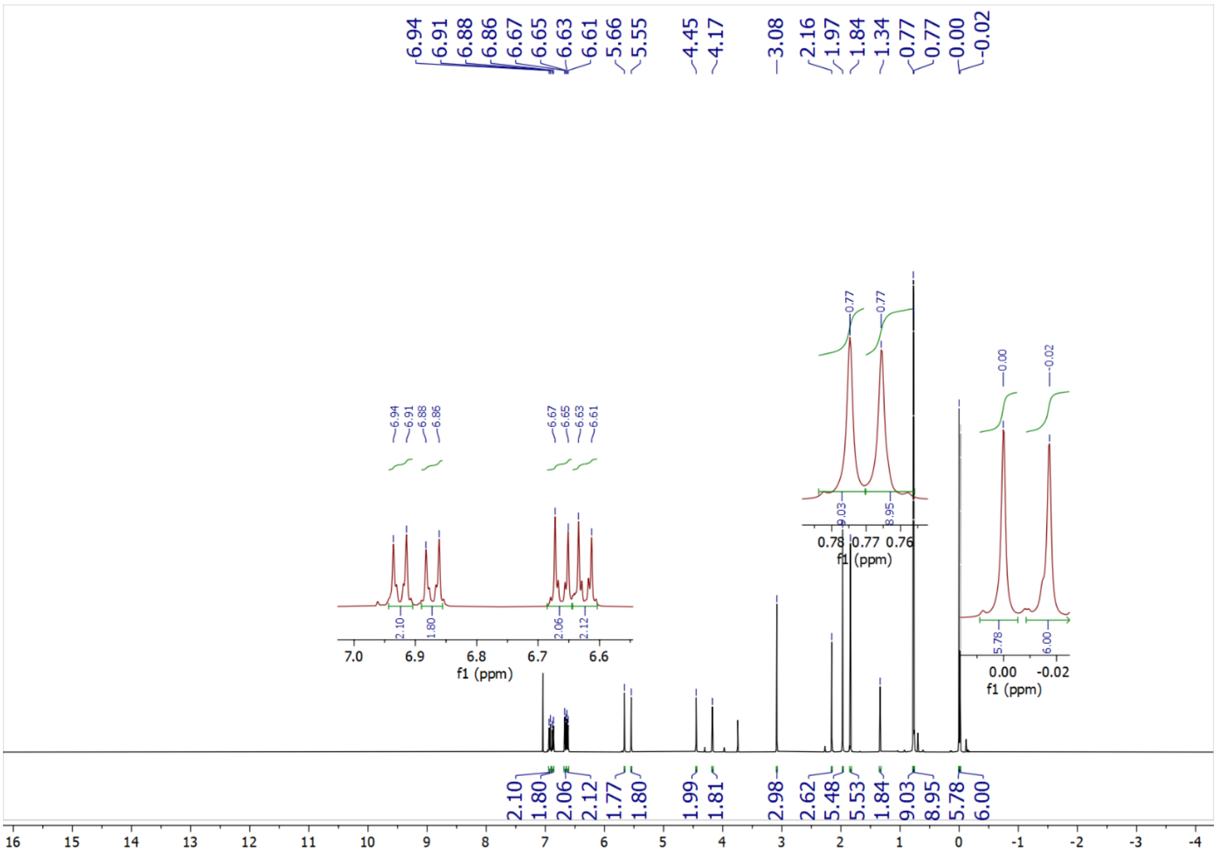

Figure S15. ^{1}H NMR, 400 MHz, $CDCl_3$ of **5**

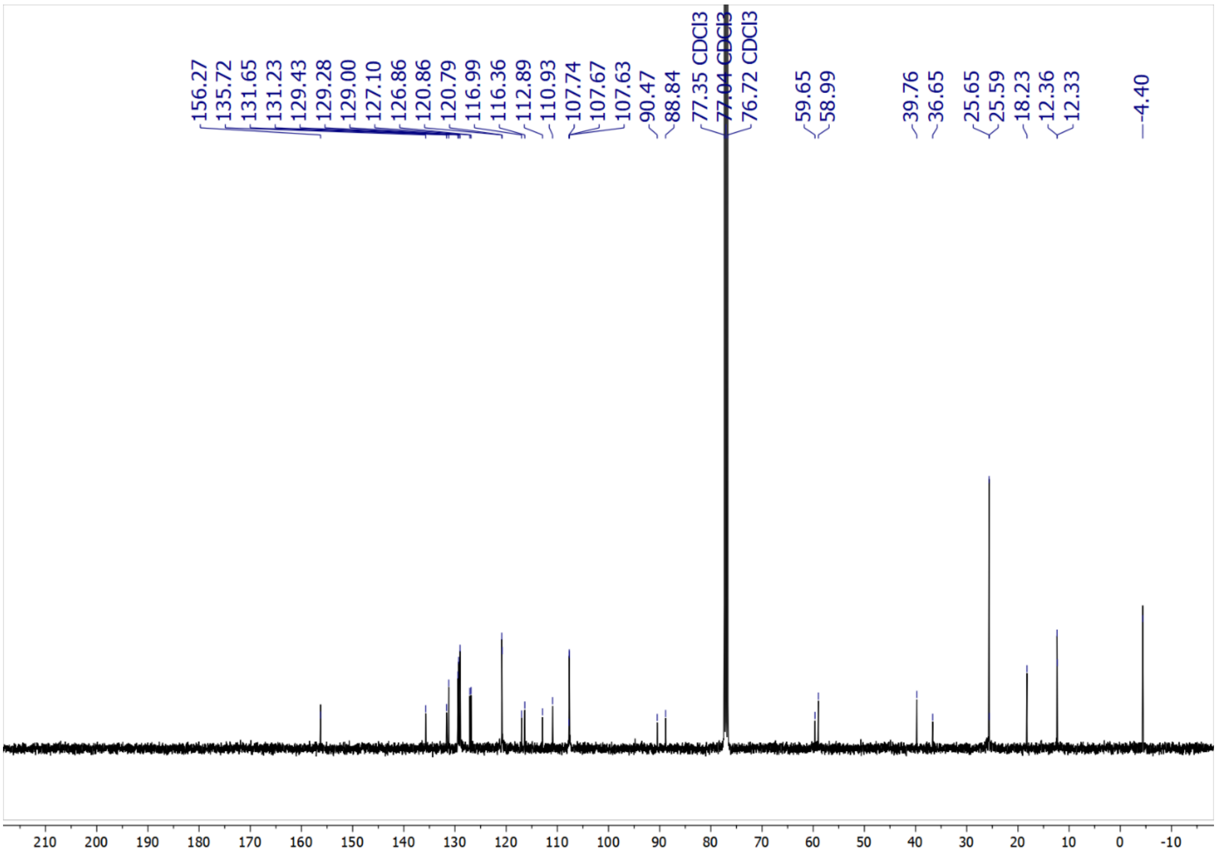

Figure S16. ^{13}C NMR 100 MHz, $CDCl_3$ of **5**

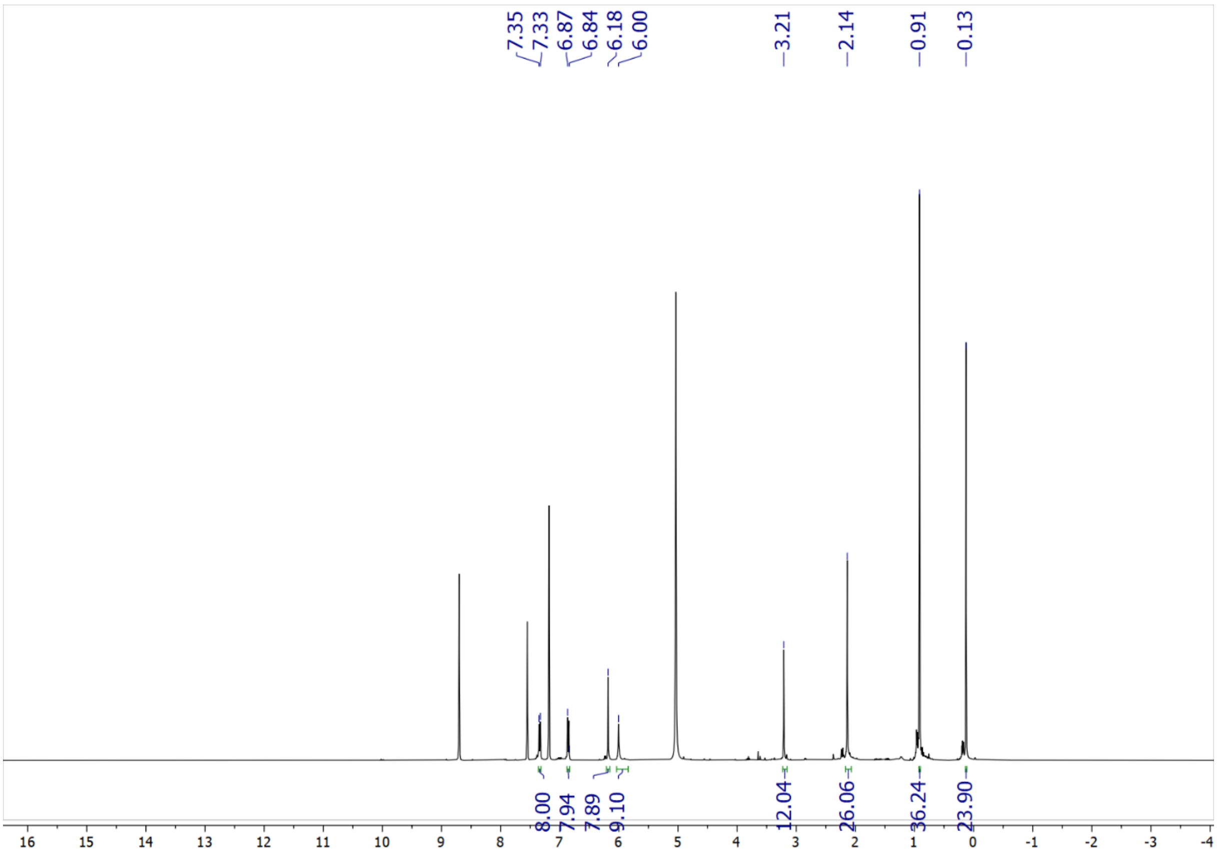

Figure S17. ^{1}H NMR, 400 MHz, pyridine-d_5 of **6**

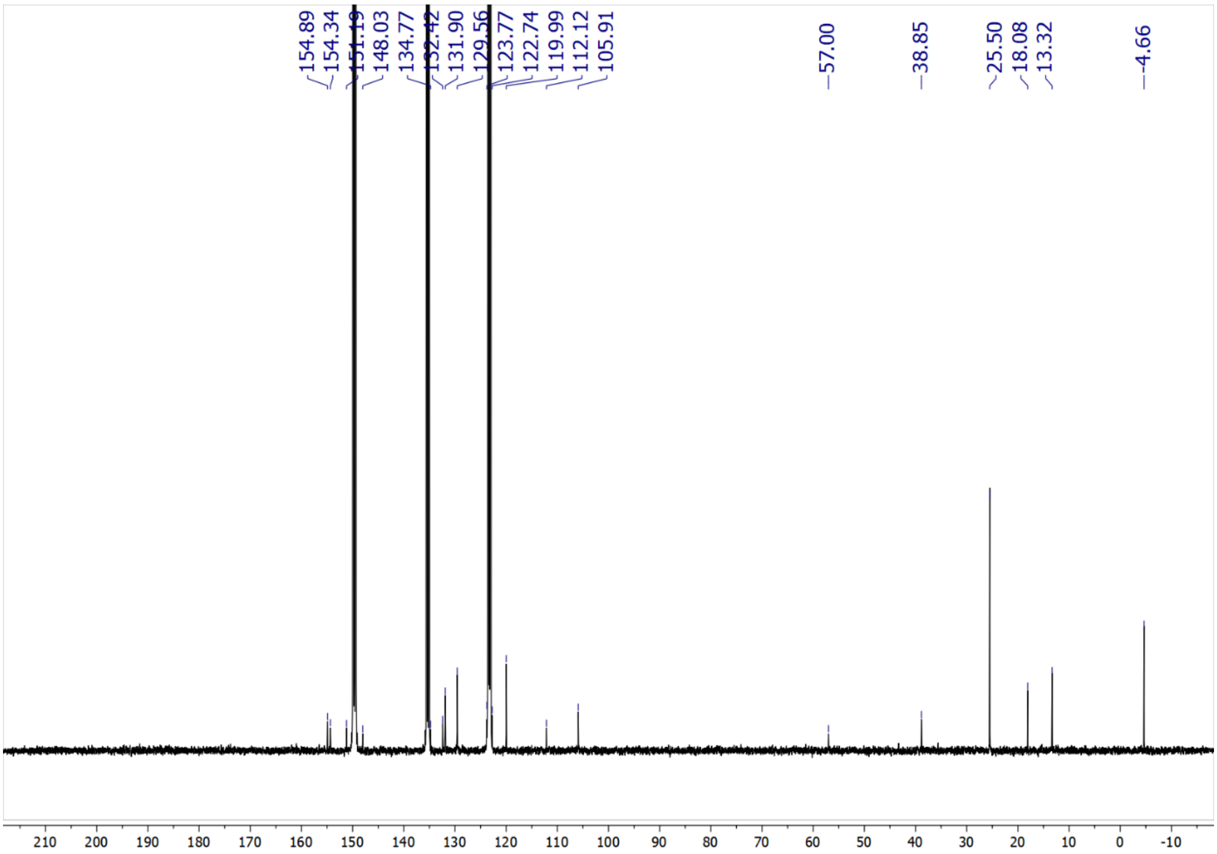

Figure S18. ^{13}C NMR 100 MHz, pyridine-d_5 of **6**

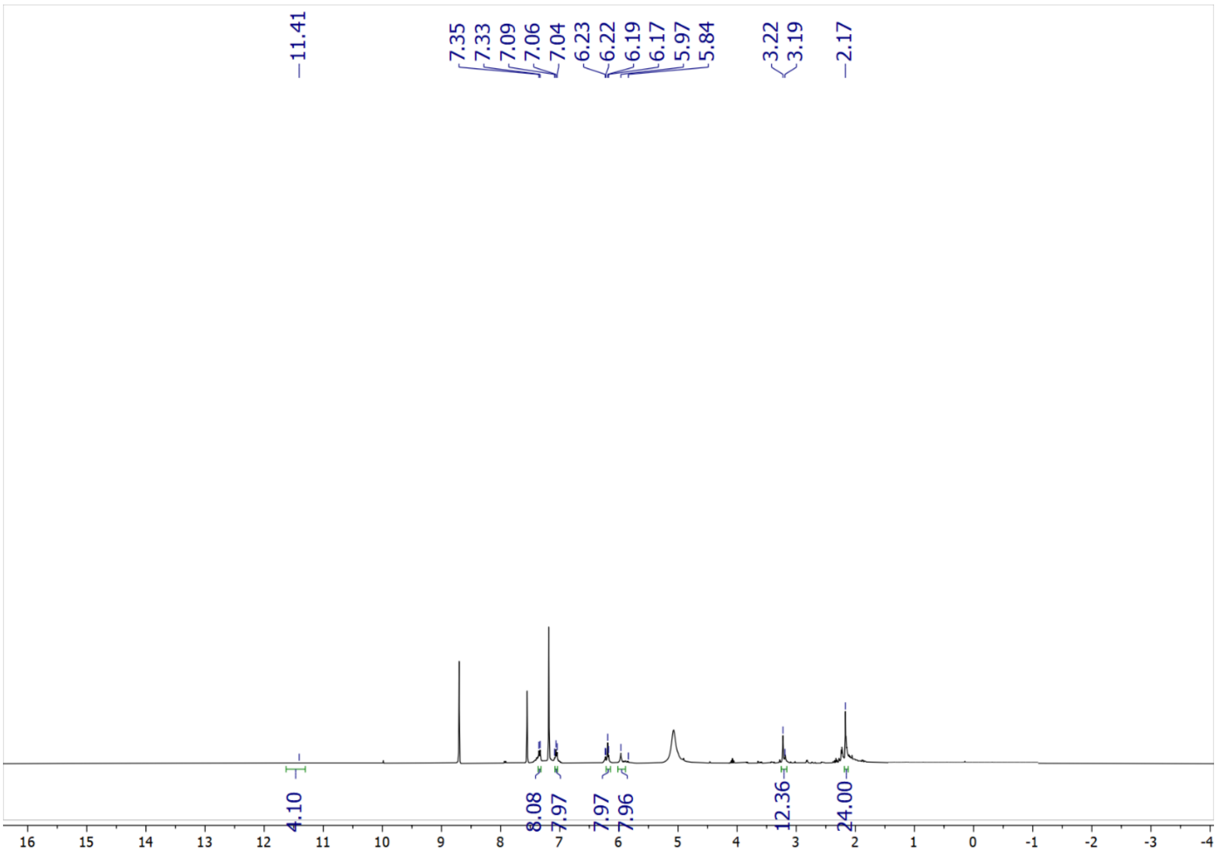

Figure S19. ^{1}H NMR, 400 MHz, pyridine-d_5 of **7**

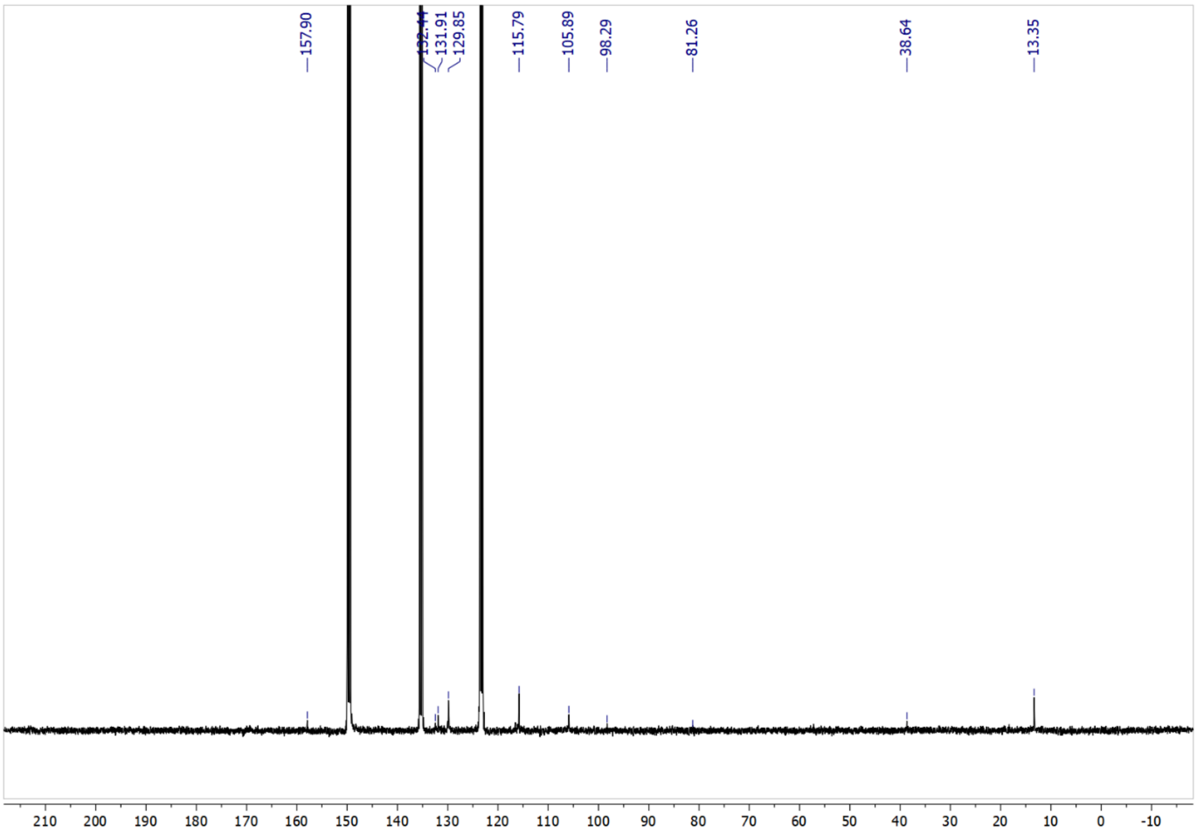

Figure S20. ^{13}C NMR 100 MHz, pyridine-d_5 of **7**

4. HRMS spectra of the compounds

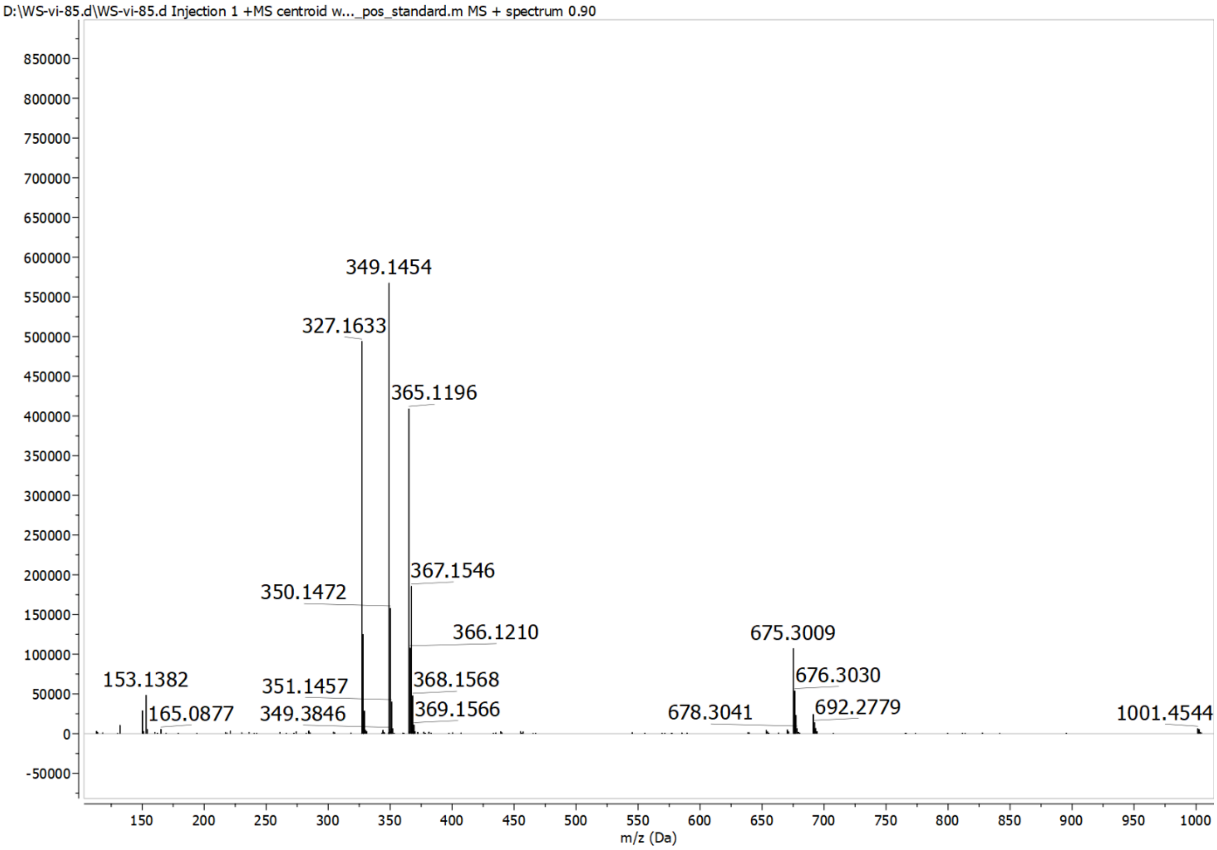

Figure S21. HMRS of **2**

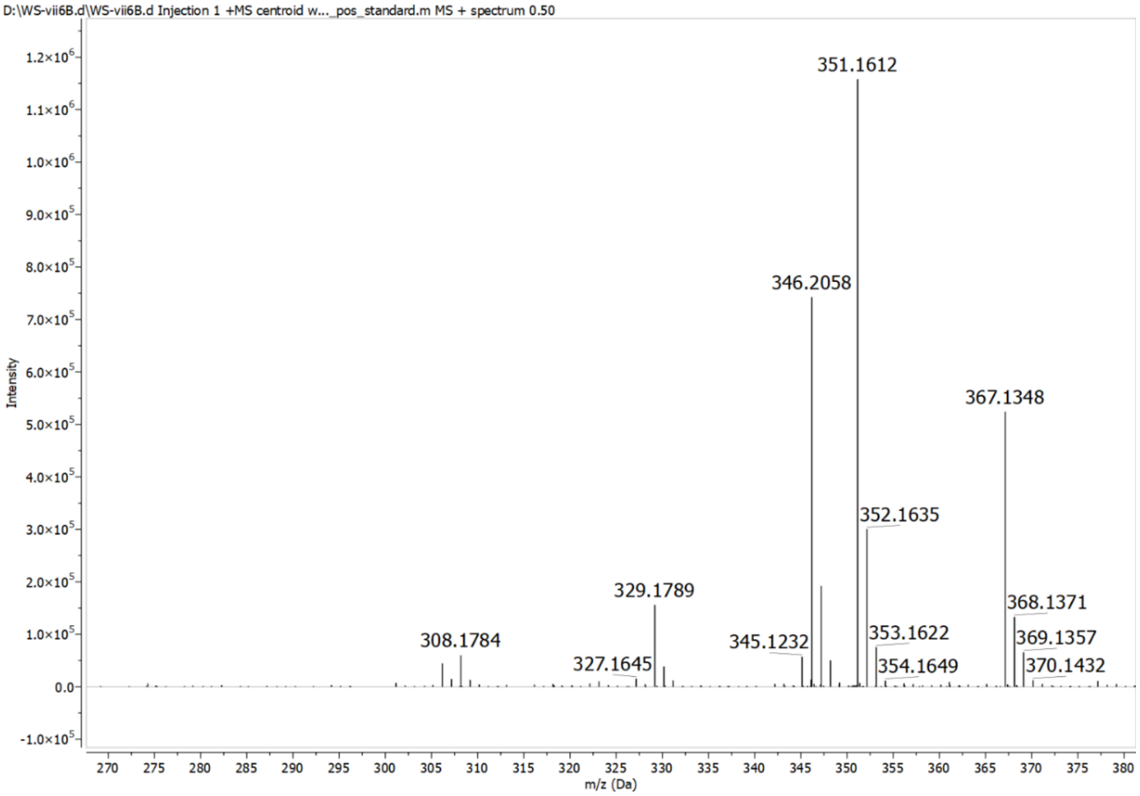

Figure S22. HRMS of **3**

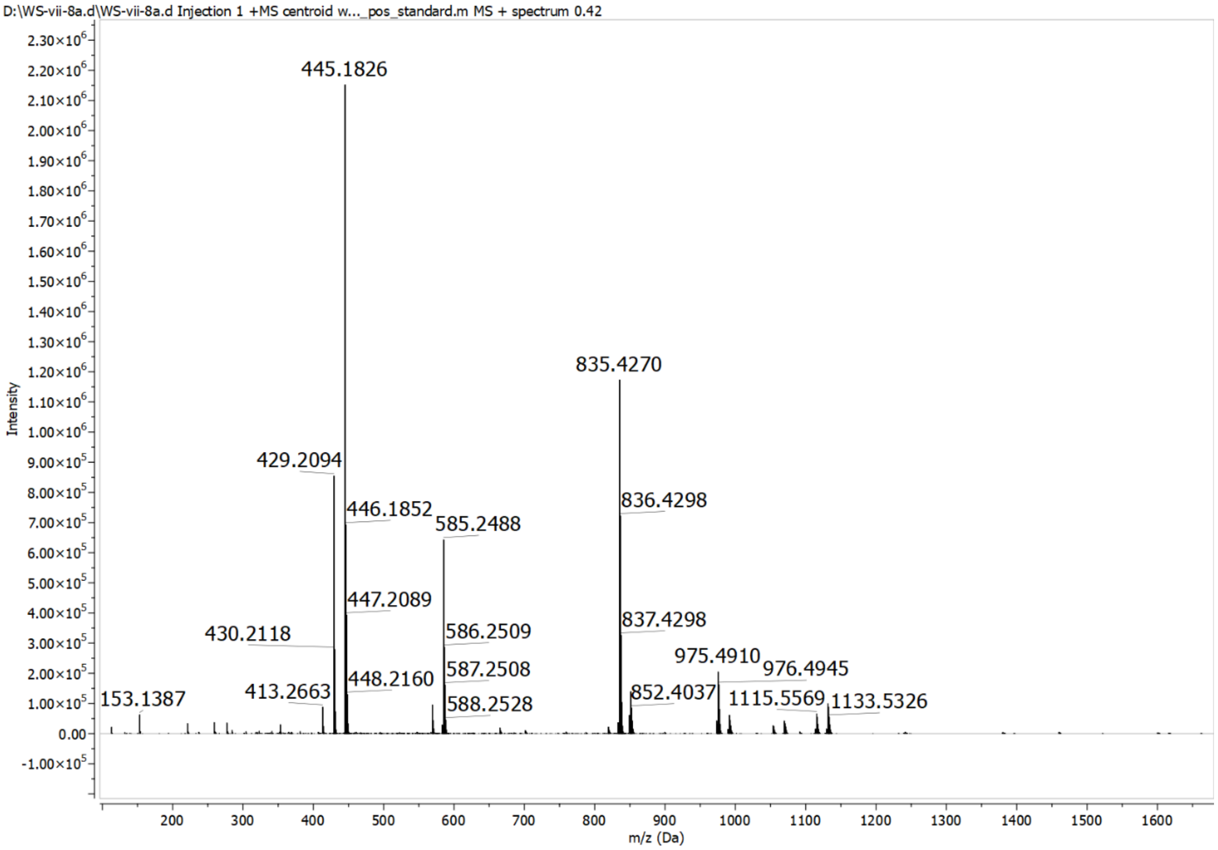

Figure S23. HRMS of **4**

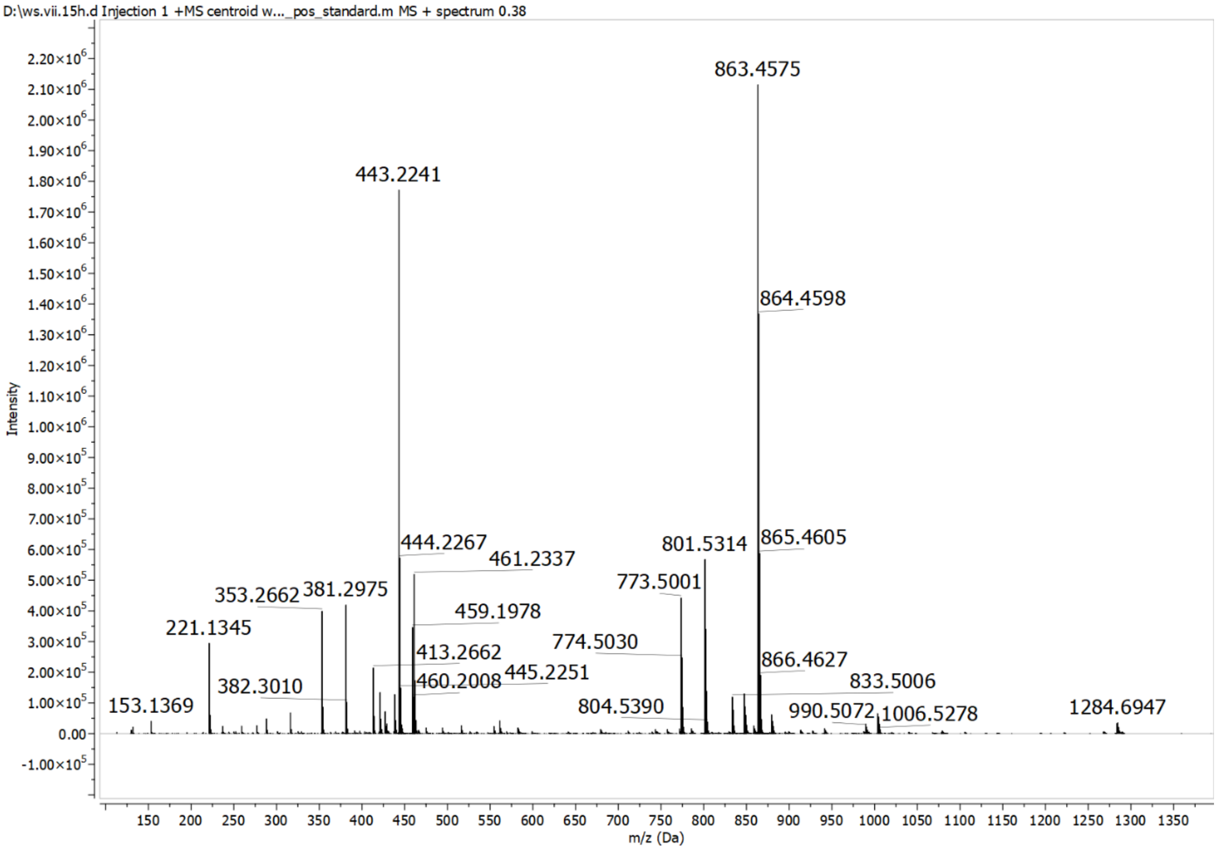

Figure S24. HRMS of **5**

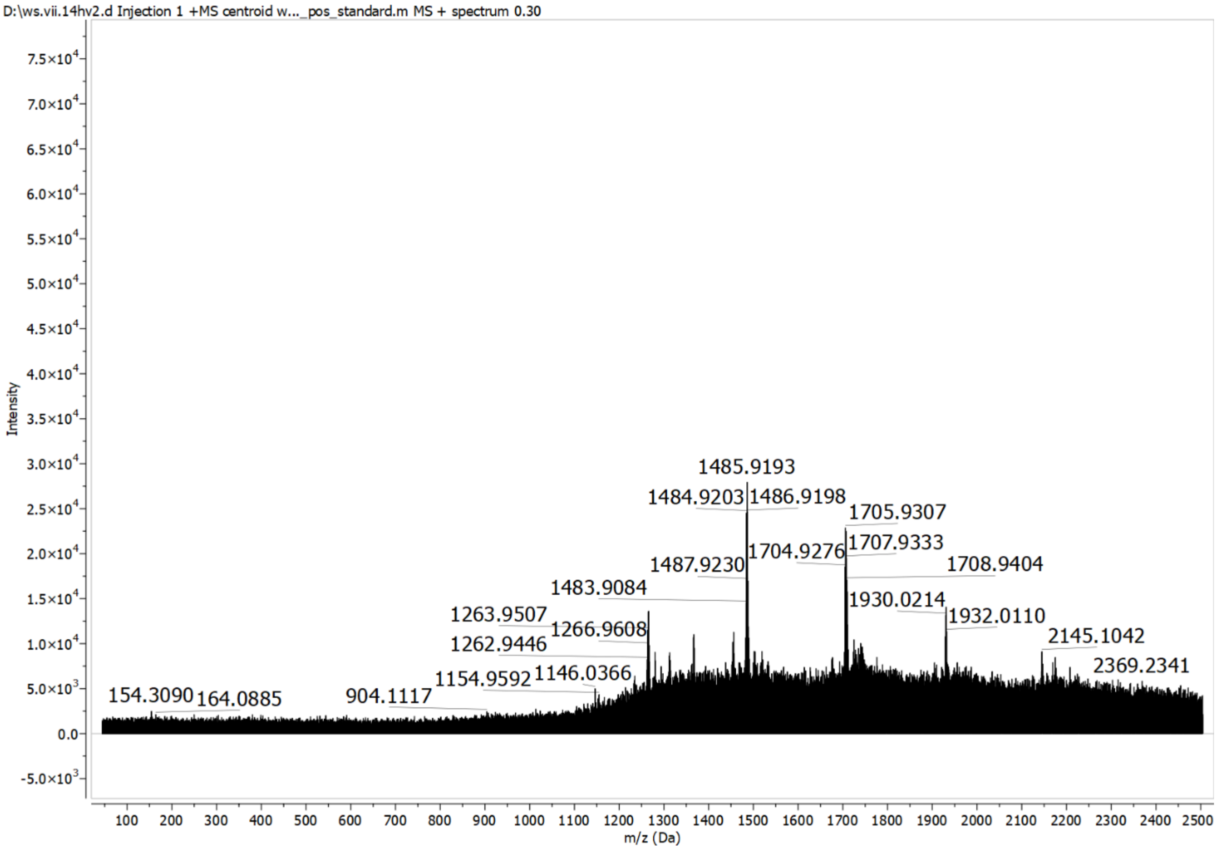

Figure S25. HRMS of **6**

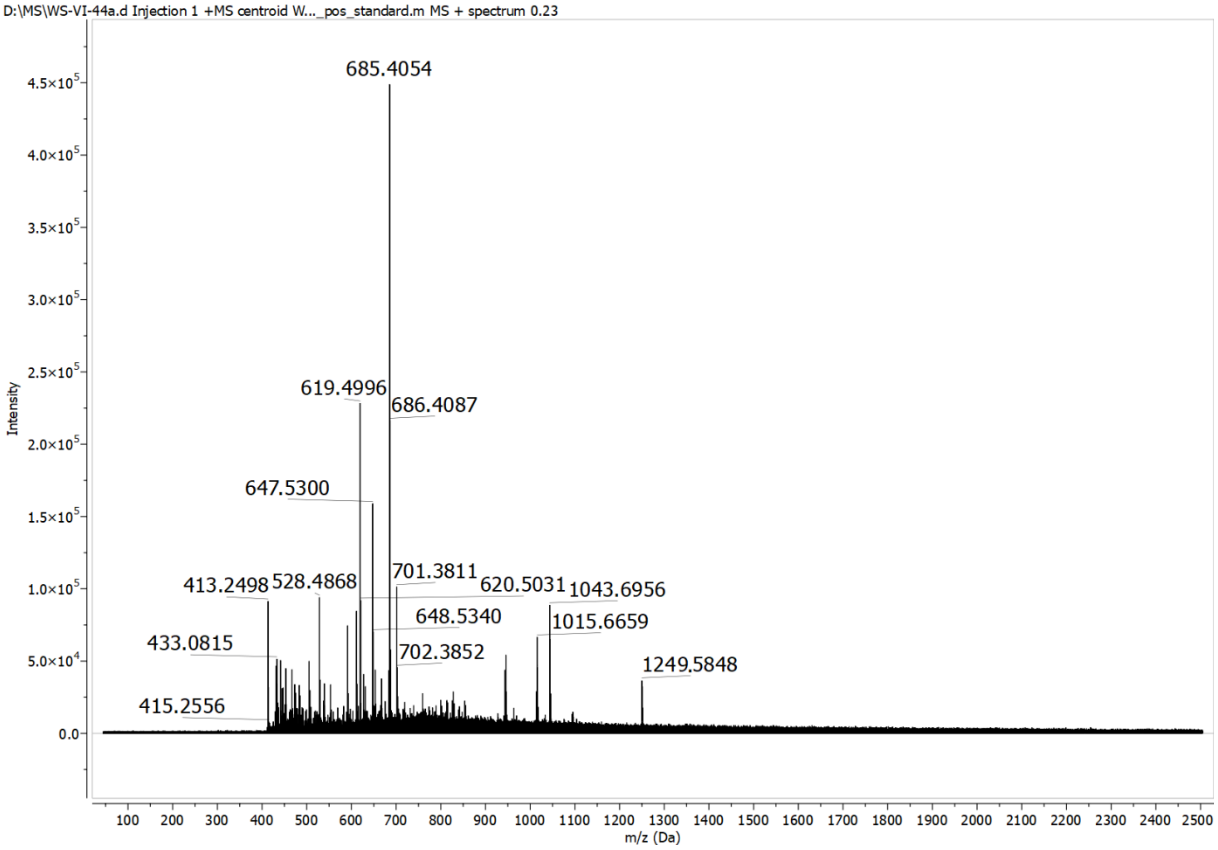

Figure S26. HRMS of **7**
